# Sonographic Evaluation of Peripheral Nerves and Cervical Nerve Roots in Amyotrophic Lateral Sclerosis: A Systematic Review and Meta-Analysis

**DOI:** 10.3390/medsci13020067

**Published:** 2025-06-01

**Authors:** Anas Elgenidy, Ibrahim A. Hassan, Yasser Hamed, Hassan Ahmed Hashem, Osama Abuel-naga, Hazem I. Abdel-Rahman, Kawashty R. Mohamed, Belal Mohamed Hamed, Mennatullah A. Shehab, Mohamed Zeyada, Somaia Kassab, Shaimaa Sabri Abdelkarim Abdelgawad, Abdelbaki Idriss Ibrahim, Ekram Hassan Hasanin, Amira A. Elhoufey, Khalid Hashim Mahmoud, Khaled Saad

**Affiliations:** 1 Faculty of Medicine, Cairo University, Cairo 12613, Egypt; 2 Faculty of Medicine, Suez Canal University, Suez 41522, Egypt; 3 Department of Neurology, Faculty of Medicine, Al-Azhar University, Assiut 71111, Egypt; 4 Department of Radiology, Faculty of Medicine, Ain Shams University, Cairo 11591, Egypt; 5 Faculty of Medicine, Al-Azhar University, Cairo 11884, Egypt; 6 Faculty of Medicine, Kafr El-Sheikh University, Kafr El-Sheikh 33516, Egypt; 7 Faculty of Medicine, Mansoura University, Mansoura 35516, Egypt; 8 Faculty of Medicine, University of Tripoli, Tripoli 13932, Libya; 9 Department of Community Health Nursing, Alddrab University College, Jazan University, Jazan 45142, Saudi Arabia; 10 Department of Community Health Nursing, Faculty of Nursing, Assiut University, Assiut 71516, Egypt; 11 Department of Pediatrics, Faculty of Medicine, Shaqra University, Dawadmi 11911, Saudi Arabia; 12 Pediatric Department, Faculty of Medicine, Assiut University, Assiut 71516, Egypt

**Keywords:** amyotrophic lateral sclerosis, peripheral nerves, cervical nerve roots, meta-analysis, ultrasound

## Abstract

Background: Amyotrophic Lateral Sclerosis (ALS) is a neurodegenerative disease that leads to nerve atrophy. Ultrasonography has a significant role in the diagnosis of ALS. Aim: We aimed to sonographically assess the size of all peripheral nerves and cervical nerve roots in ALS compared to controls. Methods: We searched MEDLINE (PubMed), Web of Science, Cochrane Central Register of Controlled Trials (CENTRAL), Embase, and Scopus using comprehensive MeSH terms for the keywords nerve, ultrasound, and ALS. We extracted data regarding cross-sectional area (CSA) or diameter for the following nerves: vagus, phrenic, tibial, fibular, sural, radial, ulnar, and median nerves, and the roots of C5, C6, C7, and C8 in both ALS patients and controls. Results: Our study included 2683 participants, of which 1631 were ALS patients (mean age = 60.36), 792 were healthy controls (mean age = 57.79), and 260 were patients with other neurological disorders. ALS patients had significantly smaller nerve size compared to controls. Nerve size differences were observed in the vagus nerve [MD = −0.23], phrenic nerve [MD = −0.25], C5 nerve root [SMD = −0.94], C6 nerve root [SMD = −1.56], C7 nerve root [SMD = −1.18], C8 nerve root [MD = −1.9], accessory nerve [MD = −0.32], sciatic nerve [MD = −11], tibial nerve [MD = −0.68], sural nerve [MD = −0.32,], ulnar nerve [MD = −0.80], and median nerve [MD = −1.21]. Conclusions: Our findings showed that ALS patients have a sonographically smaller nerve size than healthy controls. Therefore, this is a potential marker for neuronal diseases.

## 1. Introduction

Amyotrophic Lateral Sclerosis (ALS), commonly called Lou Gehrig’s disease, is a progressive neurodegenerative condition that impacts upper and lower motor neurons (UMNs and LMNs), leading to nerve atrophy and muscular weakening [1,2]. The global and economic burden of ALS in the USA is substantial, with national costs estimated to range from $279 million to $472 million [3].

ALS impacts UMNs and LMNs, causing nerve atrophy and muscular weakening [1]. The disease progressively causes weakness and subsequent paralysis of facial and bulbar muscles, as well as extremities, including the diaphragm muscles. This progression leads to respiratory failure, which is considered the primary cause of death in ALS, especially in its advanced stages [1].

Nerve atrophy in ALS affects multiple nerves, including the vagus, phrenic, and accessory nerves; cervical roots such as C5, C6, and C7; and peripheral nerves such as the ulnar, median, tibial, sural, and fibular nerves [4]. These nerve atrophies are related to the clinical symptoms of ALS, particularly respiratory impairment caused mainly by phrenic nerve atrophy, autonomic instability in vagal atrophy, and motor weakness due to peripheral nerve atrophy [4,5].

Ultrasonography (US) has demonstrated its potential in the diagnosis and differentiation of various motor neuron diseases, including amyotrophic lateral sclerosis (ALS) and its variants, as well as Charcot-Marie-Tooth disease (CMT), allowing for in vivo detection of peripheral nerve alterations, which can be crucial in distinguishing between these conditions, especially when clinical differentiation is challenging [6].

Diagnosing ALS is primarily clinical, relying on the identification of specific signs of motor neuron lesions and the exclusion of other conditions [7]. The diagnostic delay in ALS is significant, with patients experiencing symptom delay of about 10–16 months from onset to the time of diagnosis, highlighting the need for more diagnostic modalities to shorten the time to diagnosis [7,8].

The nerve cross-sectional area (CSA) detected by the US has been around for a while and has developed into a diagnostic marker, especially for peripheral neuropathies or compression injuries [9].

CSA enlargement is frequently associated with both CMT and chronic inflammatory demyelinating polyradiculopathy (CIDP); yet, unaffected CSA levels have also been reported, depending on the disease stage and the genetic background [10]. For instance, compared to treated individuals, untreated CIDP patients had larger CSAs, which is a sign of increased disease activity. Our systematic review and meta-analysis aimed to thoroughly assess the peripheral nerves and nerve roots in ALS patients compared to controls using ultrasound.

## 2. Methodology

### 2.1. Materials and Methods

We performed the systematic review and meta-analysis following the PRISMA guidelines [11]. The study protocol is registered on PROSPERO with the ID CRD42024522909.

### 2.2. Search Strategy

We conducted extensive searches in several databases, including MEDLINE (PubMed), Web of Science (WOS), Cochrane Central Register of Controlled Trials (CENTRAL), Embase, and Scopus, using comprehensive and inclusive terms, including MeSH terms for keywords, such as “nerve”, “ultrasound”, and “ALS”, up to 21 March 2024. There were no restrictions on language or study design. We also reviewed literature reviews and articles to identify other eligible studies. Detailed information on our search strategy is available in (Supplementary Table S1).

Search results were collected and filtered by ENDNOTE 21^®^, and duplicates were identified and removed. The remaining articles were uploaded to an Excel sheet for title and abstract screening [12]. Two authors independently screened the studies by title and abstract according to our eligibility criteria; a third author evaluated any discrepancies. Two authors independently screened all the included full-text articles, resolving conflicts through discussion. We have also conducted a manual search by reviewing the references of the included studies and any published reviews.

### 2.3. Eligibility Criteria

The inclusion criteria:

(1) Research articles published in international peer-reviewed journals.
(2) Studies that assessed nerve cross-sectional area (CSA) or diameter using ultrasound in individuals with ALS.
(3) Studies providing numerical data for nerve measurements, detailed as the mean and standard deviation (SD) for each group (ALS or control), separately.
(4) Other studies lacking the required numerical data were incorporated solely in the systematic review.

The exclusion criteria:

(1) Studies that did not utilize ultrasound for measuring nerve CSA or diameter.
(2) Studies that failed to present the results of ultrasound measurements.
(3) Other study designs, e.g., literature review, case studies, case series.

### 2.4. Extraction

Two authors independently extracted data on baseline characteristics from the included studies using a standardized Excel sheet. This sheet recorded information such as the first author’s name, publication year, country, sample size, participant demographics (age and gender), duration of illness, nerves measured, type of device used for measurement, and the objectives and conclusions of each study. The same two authors independently extracted the mean CSA (mm^2^) and diameter (mm) and the corresponding standard deviation (SD) of the following nerves: vagus, phrenic, tibial, fibular, sural, radial, ulnar, and median nerves, and the roots of C5, C6, C7, and C8 in both ALS patients and controls. We used the latest data for patients with multiple follow-up measurements.

### 2.5. Quality Assessment

Two authors independently evaluated the quality of the included studies using the NIH tool. This tool is designed for observational cohort and cross-sectional studies and assesses factors such as the clarity of purpose and population, measurement of exposure, outcomes, and other confounding variables [13].

The Newcastle–Ottawa Scale (NOS) was employed for quality assessment in case-control studies. This scale evaluates three main categories: selection of the study population, comparability between study groups, and ascertainment of outcome or exposure. It has a maximum score of 9, where scores from 7 to 9 indicate high quality, 4 to 6 suggest a high risk of bias, and scores from 0 to 3 indicate a very high risk of bias [14]. The quality of the included studies was categorized as good, fair, or poor. Any disagreements between the assessors were resolved by consulting a third author.

### 2.6. Statistical Analysis

The meta-analyses were done using the R software’s meta-package (R version 4.4.0) [15,16]. We evaluated the overall estimated effect employing a random-effects model. A direct pairwise meta-analysis was conducted for studies comparing nerve CSA or diameter in ALS patients and healthy controls. A single-arm meta-analysis was also performed to estimate the pooled mean nerve size in ALS patients for studies lacking control group data. Data were extracted as mean and standard deviation; for studies that presented data as median and interquartile range, we converted these to mean and standard deviation using the methods proposed by Wan et al. (2014) [17] and Luo et al. (2018) [18].

Standardized mean difference was calculated for studies that used diameter to measure nerve size. We combined the data of ALS patients’ subgroups of the same study into one group using an online calculator [19]. Subgroup analysis was done for the participants according to side and site of measurement.

Statistical heterogeneity was evaluated by calculating the I^2^ statistic and performing a chi-squared test. An I^2^ value exceeding 50% signified considerable heterogeneity, and a *p*-value below 0.05 was deemed significant.

Sensitivity analyses were conducted using the leave-one-out approach, where each meta-analysis was repeated by sequentially excluding one study at a time. This helped to identify sources of heterogeneity and determine each study’s influence on the overall estimate. Publication bias was assessed by examining a funnel plot and conducting Egger’s test, as described in [20].

## 3. Results

### 3.1. Search Results

**Figure 1:**
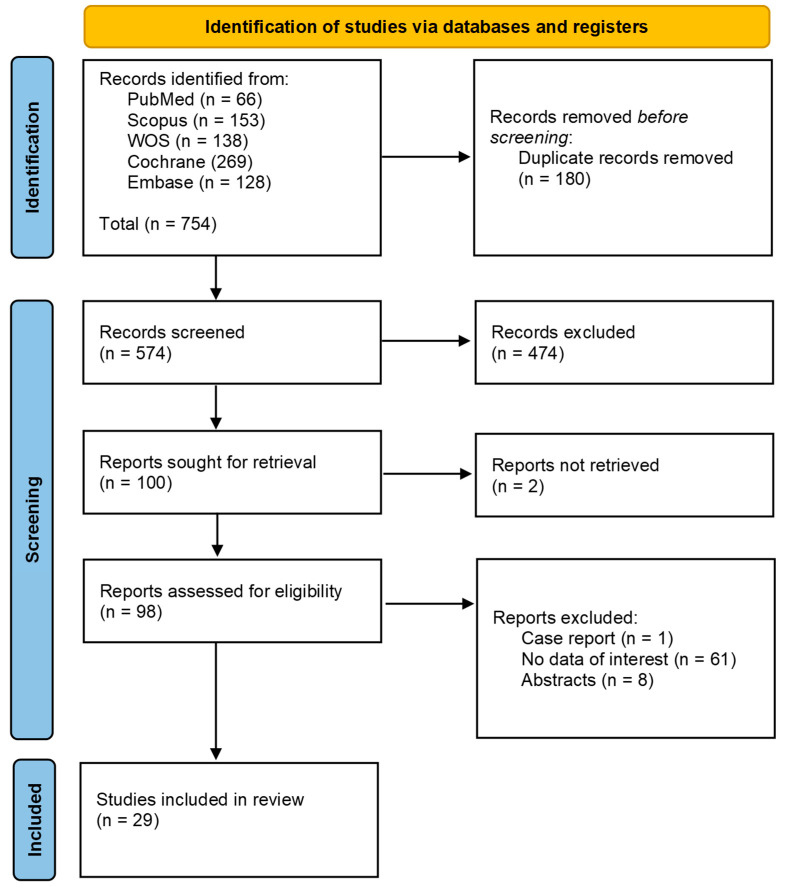
Identification of studies via databases and registers.

Our search strategy identified a total of 754 studies. After screening titles and abstracts and removing duplicates, 656 studies were excluded. We evaluated 98 full-text articles for eligibility. Following this evaluation, twenty-nine studies [21,22,23,24,25,26,27,28,29,30,31,32,33,34,35,36,37,38,39,40,41,42,43,44,45,46,47,48,49] were included in our meta-analysis, of which seven were case-control studies and 22 were cohort and cross-sectional studies. The Prisma flow diagram shows the screening process through the different phases of the study (Figure 1).

### 3.2. Summary Characteristics

Our study included 2683 participants, of which 1631 were ALS patients (mean age = 60.36), 792 were healthy controls (mean age = 57.79), and 260 were patients with other neurological disorders. The mean age of participants in all studies was about 60.36 and 57.79 for ALS patients and healthy controls, respectively.

The nerves examined include ulnar, median, radial, sciatic, sural, fibular, tibial, accessory, phrenic, vagus, and nerve roots of C5, C6, C7, and C8, unilaterally or bilaterally. The duration of ALS ranges from a low of (3.5 ± 6.25) months to a high of (53.16 ± 13.2) months. Further details regarding the type of ultrasound devices used and study characteristics are illustrated in Table 1 and Supplementary Table S2.

### 3.3. Meta-Analysis

#### 3.3.1. Ulnar Nerve

**Figure 2:**
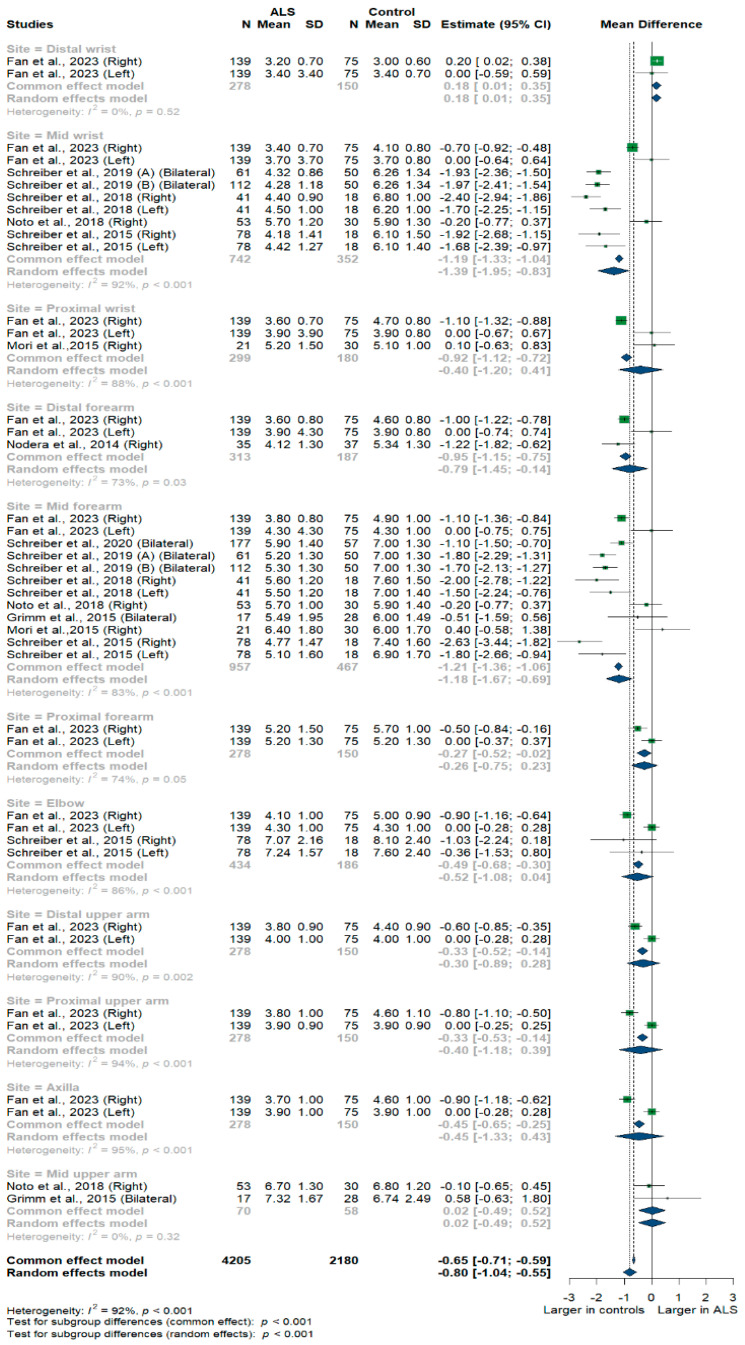
Meta-analysis of ulnar nerve cross-sectional areas in ALS patients compared to controls. CI = confidence interval; SD = standard deviation; CSA = cross-sectional area; ALS = amyotrophic lateral sclerosis. Green color = ALS patients; Blue color = healthy controls.

Nine studies and 6385 measurements comparing the cross-sectional area of the ulnar nerve with healthy controls were conducted. Across these nine studies, the ulnar nerve was assessed at 11 different sites: the distal, mid, and proximal wrist; distal, mid, proximal, and distal forearm; elbow; distal, mid, and proximal upper arm; and axilla. Median nerve size was significantly smaller in ALS patients compared to controls [MD = −0.80 mm^2^, 95%CI (−1.04 to −0.55); *p* < 0.001]. The I^2^ test showed considerable heterogeneity (I^2^ = 92%, *p* < 0.001). The test for subgroup differences came back as significant (*p* < 0.001) (Figure 2).

#### 3.3.2. Median Nerve

**Figure 3:**
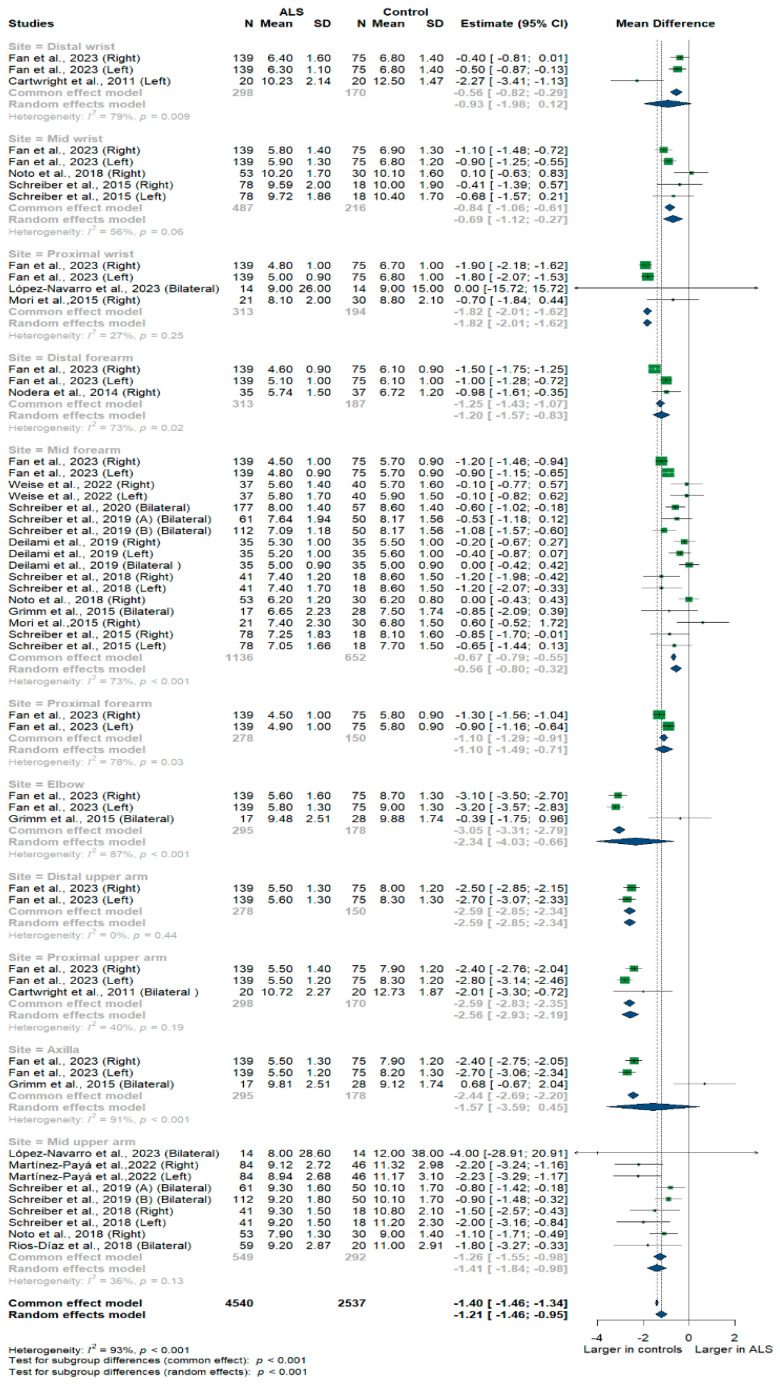
Shows the Meta-analysis of median nerve cross-sectional area in ALS patients compared to controls. CI = confidence interval; SD = standard deviation; CSA = cross-sectional area; ALS = amyotrophic lateral sclerosis; Green color = ALS patients; Blue color = healthy controls.

Fifteen studies and 7077 measurements comparing the cross-sectional area of the median nerve with healthy controls were conducted. Across these nine studies, the median nerve was assessed at 11 different sites: the distal, mid, and proximal wrist, distal, mid, and proximal forearm, elbow, distal, mid, and proximal upper arm, and axilla. Median nerve size was significantly smaller in ALS patients compared to controls [MD = −1.21, 95%CI (−1.46 to −0.95); *p* < 0.001]. The overall I^2^ test showed significant heterogeneity (I^2^ = 93%, *p* < 0.001); the test for subgroup differences came back as significant (*p* < 0.001) (Figure 3).

#### 3.3.3. Vagus Nerve

**Figure 4:**
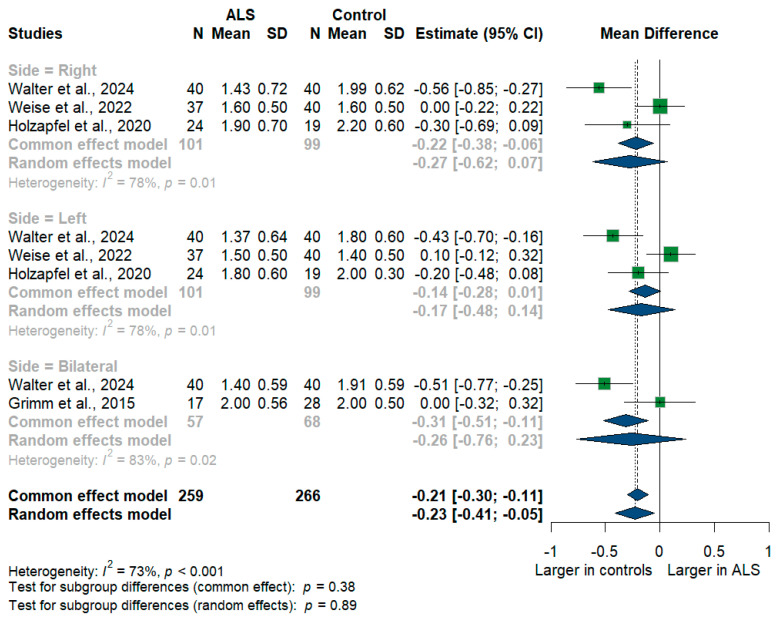
Meta-analysis of vagus nerve cross-sectional area in ALS patients compared to controls. CI = confidence interval; SD = standard deviation; ALS = amyotrophic lateral sclerosis; Green color = ALS patients; Blue color = healthy controls.

We had four studies with 525 measurements comparing the cross-sectional area of the vagus nerve with healthy controls. Vagus nerve size was significantly smaller in ALS patients compared to controls with [mean difference (MD) = −0.23 mm^2^, 95%CI (−0.41 to −0.05); *p* < 0.001]. The I^2^ test showed substantial heterogeneity (I^2^ = 73%, *p* < 0.001) (Figure 4). The studies were subgrouped according to the measurement side. Weise et al. [46] and Holzapfel et al. [26] reported right and left measurements, while Grimm et al. [22] reported only the average of bilateral measurements, and Walter et al. [45] reported right, left, and bilateral measurements. Furthermore, the right (RT) vagus nerve size was significantly smaller in ALS patients compared to controls with [MD = −0.27, 95%CI (−0.62 to −0.07); *p* < 0.001)], the left (LT) vagus nerve size was smaller in ALS patients with [MD = −0.17, 95%CI (−0.48 to 0.14); but was not statistically significant, *p* > 0.05], and the bilateral vagus nerve size was smaller in ALS patients with [MD = −0.26, 95%CI (−0.76 to 0.23); but was not statistically significant, *p* > 0.05].

#### 3.3.4. Phrenic Nerve

**Figure 5:**
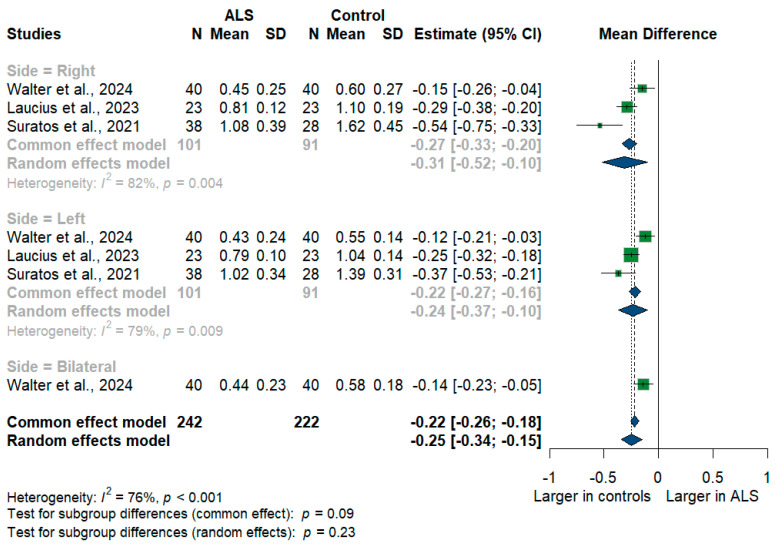
Meta-analysis of phrenic nerve cross-sectional area in ALS patients compared to controls. CI = confidence interval; SD = standard deviation; ALS = amyotrophic lateral sclerosis; Green color = ALS patients; Blue color = healthy controls.

We had three studies with 464 measurements comparing the cross-sectional area of the phrenic nerve with healthy controls. The studies were subgrouped according to the measurement side. Phrenic nerve size was significantly smaller in ALS patients compared to controls with [MD = −0.25, 95%CI (−0.34 to −0.15); *p* < 0.001] (Figure 5). Furthermore, the RT phrenic nerve size was significantly smaller in ALS patients compared to controls with [MD = −0.31, 95%CI (−0.52 to −0.10); *p* < 0.001)], the LT phrenic nerve size was smaller in ALS patients with [MD = −0.24, 95%CI (−0.37 to 0.10); *p* < 0.001], and the bilateral phrenic nerve size was smaller in ALS patients with [MD = −0.14, 95%CI (−0.23 to −0.05); *p* < 0.001]. The overall I^2^ test showed substantial heterogeneity (I^2^ = 76%, *p* < 0.001).

#### 3.3.5. C5 Root

**Figure 6:**
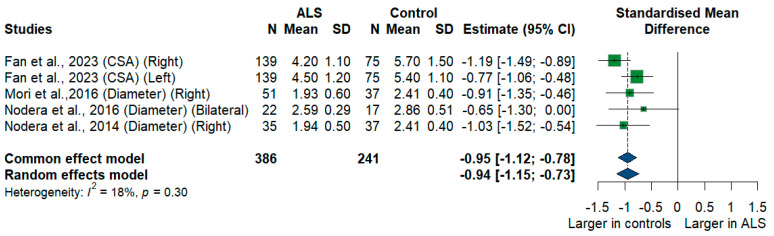
Meta-Analysis of C5 root size in ALS patients compared to controls. CI = confidence interval; SD = standard deviation; CSA = cross-sectional area; ALS = amyotrophic lateral sclerosis; Green color = ALS patients; Blue color = healthy controls.

We had four studies with 627 measurements comparing the cross-sectional area or diameter of the C5 root of ALS patients with healthy controls. C5 root size was significantly smaller in ALS patients compared to controls [SMD = −0.94, 95%CI (−1.15 to −0.73); *p* < 0.001]. The I^2^ test showed insignificant heterogeneity (I^2^ = 18%, *p* = 0.30) (Figure 6).

#### 3.3.6. C6 Root

**Figure 7:**
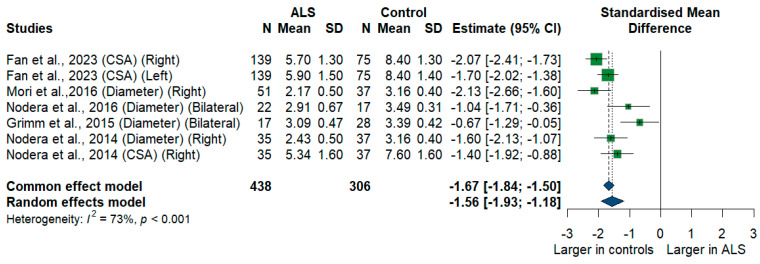
Meta-analysis of C6 root size in ALS patients compared to controls. CI = confidence interval; SD = Standard deviation; CSA = cross-sectional area; ALS = amyotrophic lateral sclerosis; Green color = ALS patients; Blue color = healthy controls.

We had five studies with 744 measurements comparing the cross-sectional area or diameter of the C6 root of ALS patients with healthy controls. C6 root size was significantly smaller in ALS patients compared to controls [SMD = −1.56, 95% CI (−1.93 to −1.18); *p*-value < 0.001]. The I^2^ test showed substantial heterogeneity (I^2^ = 73%, *p* < 0.001) (Figure 7).

#### 3.3.7. C7 Root

**Figure 8:**
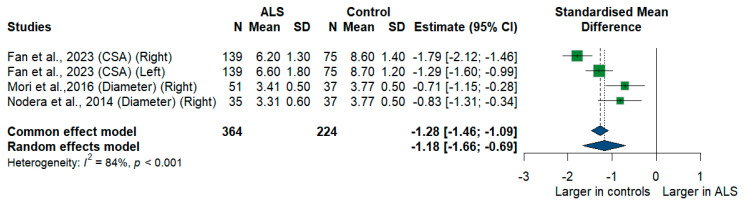
Meta-analysis of C7 root size in ALS patients compared to controls. CI = confidence interval; SD = standard deviation; CSA = cross-sectional area; ALS = amyotrophic lateral sclerosis; Green color = ALS patients; Blue color = healthy controls.

We had three studies with 588 measurements comparing the cross-sectional area or diameter of the C7 root of ALS patients with healthy controls. C7 root size was significantly smaller in ALS patients compared to controls [SMD = −1.18, 95%CI (−1.66 to −0.69); *p* < 0.001]. The I^2^ test showed substantial heterogeneity (I^2^ = 84%, *p* < 0.001) (Figure 8).

#### 3.3.8. C8 Nerve Root

We had only one study (Fan et al., 2023) [24] that compared the C8 nerve root cross-sectional area between ALS patients and healthy controls. It reported the right and left C8 nerve root cross-sectional area. The right-sided cross-sectional area was larger in controls than in ALS [MD = −1.9, 95%CI (−2.29 to −1.51); *p* < 0.001]. Similarly, the left side cross-sectional area was larger in controls than in ALS, with a difference of [MD = −2.0, 95%CI (−2.41 to −1.59); *p* < 0.001].

#### 3.3.9. Accessory Nerve

Only one study (Walter et al., 2024) [45] compared the accessory nerve cross-sectional area between ALS patients and healthy controls. It reported the right, left, and bilateral average accessory nerve cross-sectional area. The right-sided cross-sectional area was larger in controls than in [MD = −0.32, 95%CI (−0.42 to −0.22); *p* < 0.001], and the left-sided cross-sectional area was larger in controls than in ALS, with [MD = −0.31, 95%CI (−0.43 to −0.19); *p* < 0.001]. Similarly, the bilateral average cross-sectional area was larger in controls than in ALS [MD = −0.31, 95%CI (−0.40 to −0.22); *p*-value < 0.001].

#### 3.3.10. Sciatic Nerve

We had only one study (López-Navarro et al., 2024) [49] that compared the Sciatic nerve cross-sectional area between ALS patients and healthy controls. It reported only the bilateral average cross-sectional area. The bilateral cross-sectional area was higher in controls than in ALS, with a mean difference of [MD = −11, 95%CI (−115.89 to 93.89); *p* = 0.8310].

#### 3.3.11. Tibial Nerve

**Figure 9:**
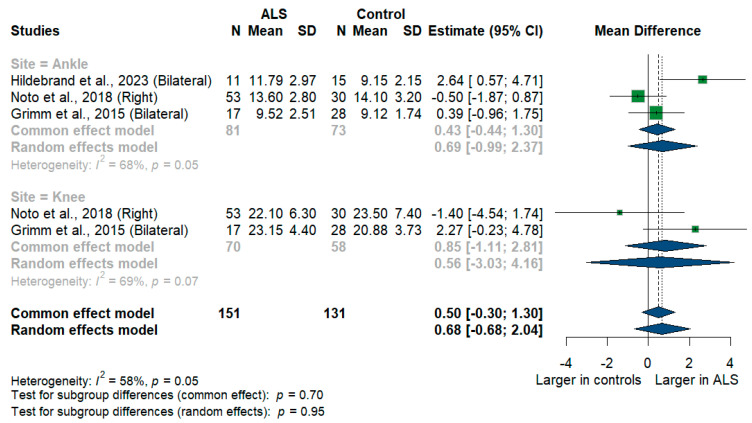
Meta-analysis of tibial nerve cross-sectional area in ALS patients compared to controls. CI = confidence interval; SD = standard deviation; CSA = cross-sectional area; ALS = amyotrophic lateral sclerosis; Green color = ALS patients; Blue color = healthy controls.

**Figure 10:**
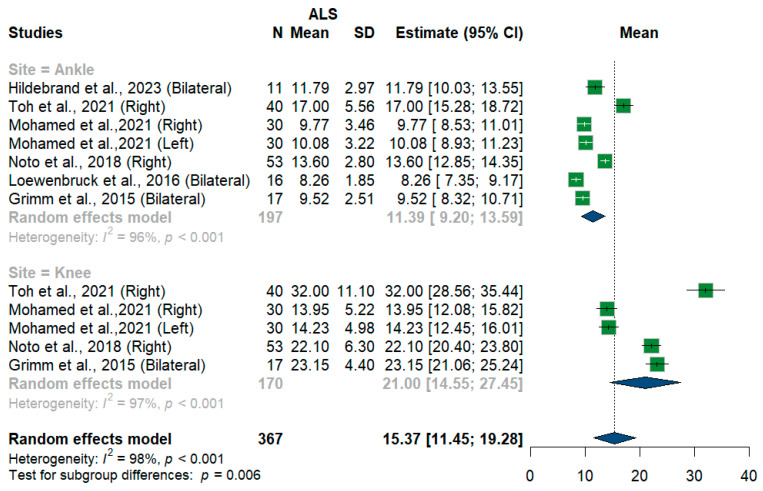
Single-arm meta-analysis of tibial nerve cross-sectional area in ALS patients. CI = confidence interval; SD = standard deviation; CSA = cross-sectional area; ALS = amyotrophic lateral sclerosis; Green color = ALS patients; Blue color = healthy controls.

We had three studies with 128 measurements comparing nerve cross-sectional areas of ALS patients with healthy controls. The studies were subgrouped according to the site where the tibial nerve was measured, whether at ankle or knee levels. Tibial nerve cross-sectional area difference was statistically insignificant between ALS patients and controls [MD = 0.68, 95%CI (−0.68 to 2.04); *p* = 0.3254]. The overall I^2^ test showed moderate heterogeneity (I^2^ = 58%, *p* = 0.05). (Figure 9). For the single-arm meta-analysis, we had six studies and 367 measurements examining the tibial nerve cross-sectional areas at the right and left knee and ankle, and other sites. The meta-analysis also showed that the pooled estimate is (15.37 mm^2^) [95%CI (11.45 to 19.28)]. The I^2^ test showed considerable heterogeneity (I^2^ = 98%, *p* <0.001). The tibial nerve at the knee level was (21.00 mm^2^) [95%CI (14.55 to 27.45)] with considerable heterogeneity (I^2^ = 97%, *p* < 0.001). The ankle level site was (11.39 mm^2^) [95%CI (9.20 to 13.59)] with considerable heterogeneity (I^2^ = 96%, *p* < 0.001). Testing for subgroup differences according to the level of the tibial nerve measurement came out as significant (*p* = 0.006) (Figure 10).

#### 3.3.12. Fibular Nerve

**Figure 11:**
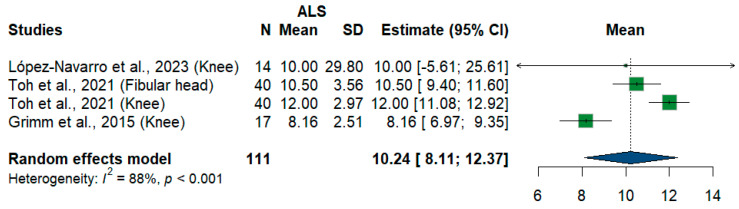
Single-arm meta-analysis of fibular nerve cross-sectional area in ALS patients. CI = confidence interval; SD = standard deviation; CSA = cross-sectional area; ALS = amyotrophic lateral sclerosis; Green color = ALS patients; Blue color = healthy controls.

We had four studies and 111 measurements assessing cross-sectional areas of the fibular nerve at different sites. The meta-analysis showed that the pooled estimate is (10.24 mm^2^) [95%CI (8.11–12.37)]. The I^2^ test showed considerable heterogeneity (I^2^ = 88%, *p* < 0.001) (Figure 11).

#### 3.3.13. Sural Nerve

**Figure 12:**
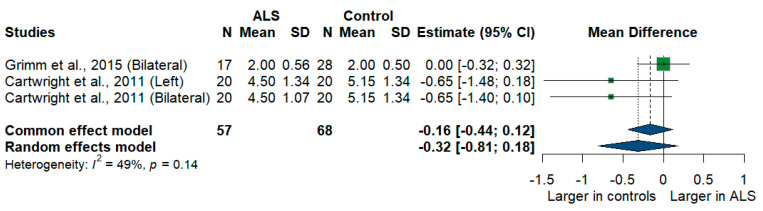
Meta-analysis of sural nerve cross-sectional area in ALS patients compared to controls. CI = confidence interval; SD = standard deviation; CSA = cross-sectional area; ALS = amyotrophic lateral sclerosis; Green color = ALS patients; Blue color = healthy controls.

Two studies compared sural nerve cross-sectional areas of ALS patients with healthy controls. The meta-analysis showed a larger sural nerve cross-sectional area in controls than in ALS patients [MD = −0.32, 95%CI (−0.81 to 0.18)], and the result was insignificant (*p* = 0.2077). The I^2^ test showed moderate heterogeneity (I^2^ = 49%, *p* = 0.14) (Figure 12).

#### 3.3.14. Radial Nerve

**Figure 13:**
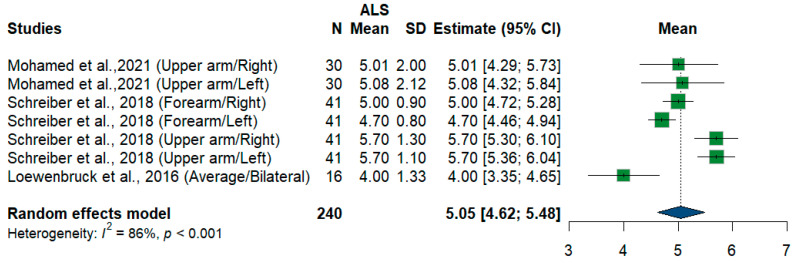
Single-arm meta-analysis of radial nerve cross-sectional area in ALS patients. CI = confidence interval; SD = standard deviation; CSA = cross-sectional area; ALS = amyotrophic lateral sclerosis; Green color = ALS patients; Blue color = healthy controls.

We had three studies and 240 measurements, assessing the cross-sectional area of the radial nerve at different sites and sides. Single-arm meta-analysis shows that the pooled mean estimate is (5.05 mm^2^) [95%CI (4.62 to 5.48)] with the overall I^2^ test, which shows considerable heterogeneity (I^2^ = 86%, *p* < 0.001) (Figure 13).

### 3.4. Leave-One-Out Meta-Analysis

Leave-one-out meta-analysis was done for all nerve measurements in the supplementary file (Figures S1–S13). It revealed one outlier in RT vagus nerve measurements as the mean difference changes to be significant upon its removal (Weise et al., 2022) [46]. Furthermore, in sural nerve measurements, upon removal of (Grimm et al., 2015) [22], the result becomes significant with larger nerve sizes in healthy controls. This might be because the participants in both studies were older than those in the other included studies. The mean age in years of Grim et al., 2015 [22] was 65 years, and the mean age in years of Weise et al., 2022, was 64 years [46].

### 3.5. Publication Bias

**Figure 14:**
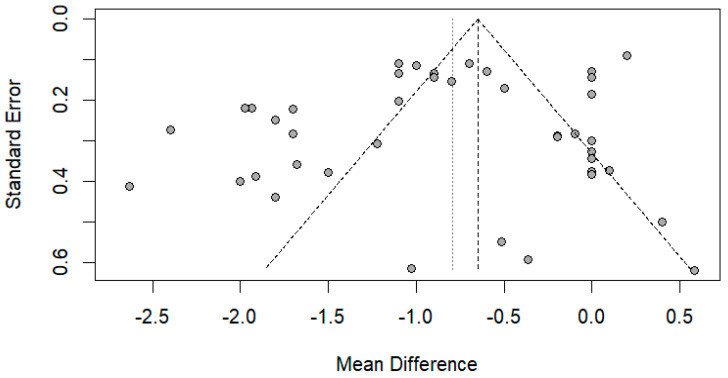
Funnel plot showing publication bias in the ulnar nerve. Egger’s test (*p*-value = 0.1212).

**Figure 15:**
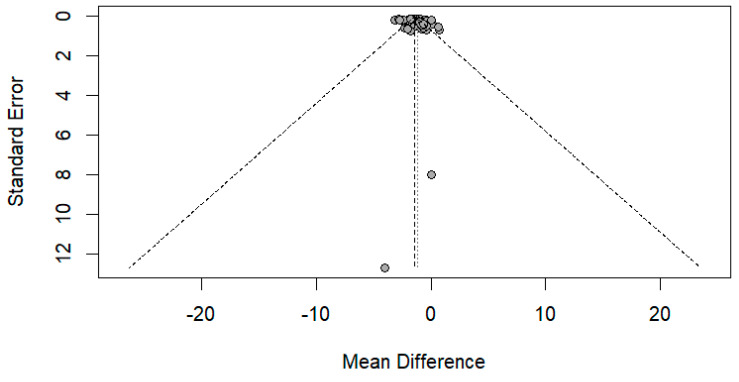
Funnel plot showing publication bias in the median nerve. Egger’s test (*p*-value = 0.1628).

The funnel plot was done for only the ulnar and median nerve results, as the other nerves included a small number of papers. After carefully assessing the funnel plots and Egger’s test results, the funnel plots revealed some asymmetry; however, Egger’s test revealed that there is no evidence of publication bias (*p*-value => 0.05) (Figure 14 and Figure 15).

### 3.6. ROC

Three studies reported diagnostic test accuracy for the US comparing nerve sizes between ALS and controls, revealing significant differences in the sensitivity and specificity of various nerves Table 2. Holzapfel et al. [26] and Schreiber et al. [38] found that the ROC curve analysis of nerve size revealed low accuracy regarding the differentiation between ALS patients and healthy controls. Furthermore, Nodera et al. [34] suggested that the ROC curves might indicate that cervical roots are more sensitive to detect axonal atrophy than the peripheral nerves. The previous results collectively suggested that the diagnostic test accuracy for the US might not be reliable for distinguishing between ALS patients and healthy controls.

### 3.7. Quality Assessment

The Newcastle–Ottawa Scale (NOS) was used to evaluate case-control studies, with five studies rated fair and two as good. For cohort and cross-sectional studies, we employed the National Institutes of Health (NIH) quality assessment tool, finding 20 studies of good quality and three of fair quality (Supplementary Tables S3 and S4).

## 4. Discussion

Our findings indicate that certain nerves exhibited a notable variation in their CSA or diameter, with a statistically significant decrease observed in the ALS group compared to the control groups, e.g., vagus nerve, phrenic nerve, C5-8 nerve roots, accessory nerve, and sciatic nerve. In contrast, others did not exhibit significant differences, e.g., tibial and sural nerves. This endorses a varied distribution of nerve involvement in ALS, which can assist in delineating and putting primary outlines about which nerves are susceptible and relevant for diagnosing ALS and tracking the disease’s progression.

In terms of motor neuron diseases, ALS is a progressive neurodegenerative condition that affects mainly motor function, manifesting as progressive weakness, wasting, and muscular paralysis, which may also lead to respiratory failure and upper and lower motor neuron signs [50].

During this disease, the corticospinal tract, brainstem, and anterior horn cells of the spinal cord show progressive neurological deterioration due to axonal degeneration and gliosis [51]. This leads to nerve atrophy, causing a reduction in both the cross-sectional area (CSA) and diameter of the nerves, which we observed in our study. Though the exact pathophysiology of ALS nerve atrophy is not fully understood, several key mechanisms have been implicated: motor neuron degeneration and glutamate excitotoxicity, as well as protein misfolding and aggregation, mitochondrial dysfunction, and neuroinflammation [52,53,54].

Typically, this nerve atrophy can be identified by its impact on nerve size. For the last decade, neuromuscular ultrasound, a high-frequency diagnostic ultrasound of peripheral nerves, has been used for this [55]. It usually takes 9–10 months after the onset of ALS symptoms to diagnose it [56].

Compared to the recent meta-analysis by Abdelnaby et al. [10], which focused on nerve sonography in Charcot-Marie-Tooth disease, our review uniquely targets ALS, emphasizing the pattern of nerve atrophy rather than enlargement. While Abdelnaby et al. provided insights into inherited neuropathies, our work highlights the diagnostic potential of ultrasound in a progressive neurodegenerative disorder, offering clinicians a new perspective on its application in ALS management.

The El Escorial and Awaji criteria have high specificity, but their sensitivity is still too low for an early diagnosis [56]. As the disease may be more responsive to treatment in its early stages, early detection and treatment can lead to better treatment outcomes [54]. The current method for diagnosing ALS uses electrodiagnostic and an expert neurological view within an appropriate clinical setting. In uncommon and inconvenient situations where ALS has been suspected but not sufficiently confirmed by standard clinical examinations and electrodiagnostic testing, nerve ultrasonography helps in the early diagnosis. An additional benefit of using U\S is that it is widely available and easily applicable, making it suitable for everyday use even in areas outside of highly specialized neuromuscular clinics. Additionally, nerve ultrasonography may be a crucial component that enhances the effectiveness of other techniques, such as muscle ultrasound, in obtaining a comprehensive image of the disease’s extent [56].

However, it was not of tremendous help because there was no agreement on diagnosing and prognosing ALS based on the specific nerves identified for it. So, there was still a knowledge gap that needed to be filled.

Our comprehensive study incorporates all the nerves we have encountered in all the articles published in this field. We had an exhaustive search strategy to encompass all the available literature discussing nerve size in ALS patients. We also used different subgrouping techniques, depending on the site or side of the nerve.

We conducted this research to assess whether nerve size in ALS patients differs from that in healthy controls. This article reported fourteen nerves: the vagus nerve, phrenic nerve, C5-8 nerve roots, accessory nerve, sciatic nerve, tibial nerve, femoral nerve, fibular nerve, radial nerve, ulnar nerve, and median nerve.

### Limitations

Some of our nerve meta-analyses revealed significant heterogeneity, which is a primary concern when interpreting the results and significance of any meta-analysis. To address this issue, we conducted a subgroup analysis based on the site or side of measurement, utilized random-effects model meta-analyses, and performed a sensitivity analysis using the leave-one-out method. Furthermore, some of our included studies measured the nerves as C5, C6, and C7 of ALS and the control in two different ways (CSA and diameter), so we used the standardized mean difference model (SMD). During our systematic review, we found limited evidence on the diagnostic test accuracy of US in ALS diagnosis, suggesting a need for further research to explore this topic more thoroughly.

## 5. Conclusions

Based on the current meta-analysis designed to distinguish ALS patients from healthy controls, our results indicate that individuals with ALS have a smaller nerve size compared to healthy controls. We concluded that the US could have a promising and futuristic role in differentiating diseases such as ALS and other neuromuscular diseases.

## Figures and Tables

**Table 1:** Summarization of meta-analyses in different nerves results.

Nerve/Subgroup	N	Sample Size		MD/SMD	95%CI	Heterogeneity	
		ALS	Control			I^2^	*p*
**Vagus**							
Total	4	259	266	−0.23	−0.41 to −0.05	73%	**<0.001**
RT	3	101	99	−0.27	−0.62 to −0.07	78%	**0.01**
LT	3	101	99	−0.17	−0.48 to 0.14	78%	**0.01**
Bilateral	2	57	68	−0.26	−0.76 to 0.23	83%	**0.02**
**Phrenic**							
Total	3	242	222	−0.25	−0.34 to −0.15	76%	**<0.001**
RT	3	101	91	−0.31	−0.52 to −0.10	82%	**0.004**
LT	3	101	91	−0.24	−0.37 to −0.10	79%	**0.009**
Bilateral	1	40	40	−0.14	−0.23 to −0.05	-	
C5 root *	4	386	241	−0.94	−1.15 to −0.73	18%	0.3
C6 root *	5	438	306	−1.56	−1.93 to −1.18	73%	**<0.001**
C7 root *	3	364	224	−1.18	−1.66 to −0.69	84%	**<0.001**
**C8 root**	1	139	75	−1.9	−1.51 to −2.29	-	-
**Accessory**	1	40	40	−0.32	−0.42 to −0.22	-	-
**Sciatic**	1	14	14	−11	−115.89 to 93.89	-	-
**Tibial**							
Total	3	151	131	−0.68	−0.68 to 2.04	58%	0.05
Ankle	3	81	73	0.69	−0.99 to 2.37	68%	0.05
Knee	2	70	58	0.56	−3.03 to 4.16	69%	0.07
**Sural**	2	57	68	−0.32	−0.81 to 0.18	49%	0.14
**Ulnar**							
Total	9	4205	2180	−0.80	−1.04 to −0.55	92%	**<0.001**
Distal wrist	2	278	150	0.18	0.01 to 0.35	0%	**0.52**
Mid wrist	9	742	352	−1.39	−1.95 to −0.83	92%	**<0.001**
Proximal wrist	3	299	180	−0.40	−1.20 to 0.41	88%	**<0.001**
Distal forearm	3	313	187	−0.79	−1.45 to −0.14	73%	**0.03**
Mid forearm	12	957	467	−1.18	−1.67 to −0.69	83%	**<0.001**
Proximal forearm	2	278	150	−0.26	−0.75 to −0.23	74%	0.05
Elbow	4	434	186	−0.52	−1.08 to 0.04	86%	**0.002**
Proximal upper arm	2	278	150	−0.40	−1.18 to 0.39	94%	**<0.001**
Distal upper arm	2	278	150	−0.30	−0.89 to 0.28	90%	**0.002**
Mid upper arm	2	70	58	0.02	−0.49 to 0.52	0%	0.32
Axilla	2	278	150	−0.45	−1.33 to 0.43	95%	**<0.001**
**Median**							
Total	15	4540	2537	−1.21	−1.46 to −0.95	93%	**<0.001**
Distal wrist	3	298	170	−0.93	−1.98 to 0.12	79%	**0.009**
Mid wrist	5	478	216	−0.69	−1.12 to −0.27	56%	**0.06**
Proximal wrist	4	313	194	−1.82	−2.01 to −1.62	27%	0.25
Distal forearm	3	313	194	−1.20	−1.57 to −0.83	73%	**0.02**
Mid forearm	17	1136	652	−0.56	−0.80 to −0.32	73%	**<0.001**
Proximal forearm	2	278	150	−1.10	−1.49 to −0.71	78%	**0.03**
Elbow	3	295	178	−2.34	−4.03 to −0.66	87%	**<0.001**
Distal upper arm	2	278	150	−2.59	−2.85 to −2.34	0%	0.44
Proximal upper arm	3	298	170	−2.56	−2.93 to −2.19	40%	0.19
Mid upper arm	9	549	292	−1.41	−1.84 to −0.98	36%	0.13
Axilla	3	295	178	−1.57	−3.59 to 0.45	91%	**<0.001**

* Nerve was analyzed using standardized mean difference (SMD).

**Table 2:** Studies reporting ROC, sensitivity, and specificity for comparison between ALS and control nerve sizes.

Article	Nerve	AUC	Sensitivity	Specificity
Holzapfel et al., 2020 [26]	Vagus nerve (CSA)	0.69	66.7%	63.2%
Schreiber et al., 2019 [38]	Ulnar nerve wrist (CSA)	0.897	69.2%	92.5%
	Ulnar nerve forearm (CSA)	0.866	87.8%	74.1%
	Median nerve wrist (CSA)	0.625	73.5%	60.7%
	Median nerve forearm (CSA)	0.687	75.0%	57.1%
Nodera et al., 2014 [34]	C5 (Diameter)	0.77	-	-
	C6 (Diameter)	0.87	-	-
	C6 (CSA)	0.84	-	-
	C7 (Diameter)	0.74	-	-
	Median nerve (Diameter)	0.66	-	-
	Median nerve (CSA)	0.72	-	-
	Ulnar nerve (Diameter)	0.71	-	-
	Ulnar nerve (CSA)	0.76	-	-

## Data Availability

The data supporting this study’s findings are available from the corresponding author upon reasonable request.

## Supplementary Materials

The following supporting information can be downloaded at: https://www.mdpi.com/article/10.3390/medsci13020067/s1, Table S1. Table showing the results of the database search; Table S2 Characteristics of studies included in the systematic review/meta-analysis; Table S3. Case-control studies by The Newcastle-Ottawa Scale (NOS) quality assessment; Table S4. Quality assessment of Cohort/cross-sectional studies by NIH; Figure S1. Leave one out meta-analysis for All Vagus nerve measurements; Figure S2. Leave one out meta-analysis for right vagus nerve measurements; Figure S3. Leave one out meta-analysis for All Phrenic nerve measurements; Figure S4. Leave one out meta-analysis for All C5 root measurements; Figure S5. Leave one out meta-analysis for All C6 root measurements; Figure S6. Leave one out meta-analysis for All C7 root measurements; Figure S7. Leave one out meta-analysis for All Tibial nerve; Figure S8. Leave one out meta-analysis for single arm Tibial nerve measurements; Figure S9. Leave one out meta-analysis for single arm Fibular nerve; Figure S10. Leave one out meta-analysis for All Sural nerve measurements; Figure S11. Leave one out of the meta-analysis for single-arm Radial nerve; Figure S12. Leave one out meta-analysis for All Ulnar nerve measurements; Figure S13. Leave one out meta-analysis for All Median nerve. Refs. [1,2,3,4,5,6,7,8,9,10,11,12,13,14,15,16,17,18,19,20,21,22,23,24,25,26,27,28,29,30,31] are cited in SM.
