# Supplementary material for: Sonographic Evaluation of Peripheral Nerves and Cervical Nerve Roots in Amyotrophic Lateral Sclerosis: A Systematic Review and Meta-Analysis
Source: Med Sci (Basel). 2025 Jun 1;13(2):67. doi: 10.3390/medsci13020067 (PMC12194937; doi:10.3390/medsci13020067)
Supplement: Supplementary file 1 [file medsci-13-00067-s001.zip › medsci-3598673-supplementary.pdf]

Supplementary file

Table S1. Table showing the results of the database search

Database	Results	Search term
**PubMed**	66	((Ultrasound OR Echotomography OR Ultrasonic OR Sonography OR Ultrasonographic OR Echography) AND ("Amyotrophic Lateral Sclerosis" OR "Gehrig's Disease" OR "Gehrig Disease" OR "Gehrigs Disease" OR "Charcot Disease" OR "Lou Gehrig's Disease" OR "Lou-Gehrigs Disease" OR ALS OR "Lou Gehrig Disease" OR "Guam Disease")) AND (nerve)
**Scopus**	153	
**WOS**	138	
**Cochrane**	269	
**Embase**	128	
**Total** (n=754)		
**Total after duplicate removal** (n=574)		

Table S2 Characteristics of studies included in the systematic review/meta-analysis

Study	Country	Total number	Sex (M/F)	Age	Duration of illness	Nerve	Device used	Aim	Conclusion
**Laucius et al., 2023**[1]	Lithuania	ALS (23)<br>Control (23)	ALS (9/14)<br>Control (11/12)	ALS (57.3±4.9)<br>Control (55.2±10.1)	ALS (15.45 ± 10.11 months)	Phrenic nerve	Philips EPIQ 7 (r 4–18 MHz linear transducer (CE 0086))	To investigate the potential use of ultrasound imaging of the phrenic nerve as a diagnostic tool for Amyotrophic Lateral Sclerosis (ALS).	The results of this small study are particularly promising, as they suggest that sonographic findings could serve as a diagnostic tool for ALS.
**Fan et al., 2023**[2]	China	ALS (139)<br>Control (75)	ALS (75/64)<br>Control (40/35)	ALS (53.36 ±11.98)<br>Control (50.55±11.7)	ALS (15.14 ± 12.64 months)	Median nerve<br>Ulnar nerve<br>brachial plexus C5, C6, C7, C8 nerve roots<br>Brachial plexus upper, medial, and lower trunks	Ultrasonography (8-MHz to 12-MHz linear-array transducer)	To determine the CSA of peripheral nerves in patients with ALS.	Peripheral nerves in ALS become progressively thinner, and ultrasound could be sensitive to axonal degeneration.
**Hildebrand et al., 2023**[3]	Germany	ALS (11)<br>CIDP (5)<br>CMT (5)<br>Control (15)	ALS (7/4)<br>CIDP (3/2)<br>CMT (4/1)<br>Control (7/8)	ALS (61.7 ±9.5)<br>CIDP (56.2 ±7.3)<br>CMT (29.8 ±8.6)<br>Control (57.6 ±22.6)	ALS (33.17 ± 52.06 months)<br>CIDP (58.17 ± 48.19 months)<br>CMT (192.03 ± 62.39 months)	Tibial nerve	High-resolution US (Philips Medical System, Affinity 70G, with an eL18-4 18-MHz)	To use peripheral nerve ultrasound to differentiate between amyotrophic lateral sclerosis (ALS), chronic inflammatory demyelinating polyradiculopathy (CIDP), Charcot-Marie-Tooth disease (CMT), and controls.	CSA and flow may potentially uncover an ALS disease subtype with more pronounced peripheral nerve inflammation and better sustained clinical function.
**Walter et al., 2023**[4]	Germany	ALS (40)<br>Control (40)	Als (26/14)<br>Controls (21/19)	ALS (66.2?±?10.5)<br>Control (66.0 ±? 8.6)	ALS (18.1 ± 14.5 months)	Vagus nerve<br>Accessory nerve<br>Phrenic nerve	HRUS, equipped with a 15.0-MHz transducer (LA435) was applied	To determine whether PN atrophy is related to respiratory function and the 12-month outcome of ALS patients, The degree and clinical correlates of spinal accessory nerve (AN) and VN atrophy in ALS.	Ultrasonography detects degeneration of cranial nerve motor fibers. Phrenic and Accessory nerve calibers are tightly related to respiratory function and 1-year survival in ALS.
**Watanabe et al., 2022**[5]	Japan	ALS (9)<br>SBMA (11)	ALS (9/0)<br>SBMA (11/0)	ALS (62.1± 17.5)<br>SBMA (66.8 ±12.5)	ALS (37.2 ± 28.8 months)<br>SBMA (169.2 ± 67.2 months)	Median nerve<br>Ulnar nerve<br>C5, C6, and C7 nerve roots	Aplio MX ultrasound system, equipped with a 13- to 18-MHz linear array transducer	To evaluate cervical nerve roots and peripheral nerves using ultrasonographic techniques in patients with SBMA and to compare the results with those in patients with ALS.	US showed that the peripheral nerves in patients with SBMA were thinner than those in patients with ALS despite similar degrees of weakness and motor neuron loss.
**Martínez-Payá et al., 2022**[6]	Spain	ALS cohort A (27)<br>ALS cohort B (57)<br>Control (64)	ALS cohort A (18/9)<br>ALS cohort B (33/24)<br>Control (27/19)	ALS cohort A (60.65 ± 9.76)<br>ALS cohort B (63.62 ± 11)<br>Control (59.9 ± 8.08)	ALS cohort A (20.57 ± 20.74 months)<br>ALS cohort B (13.78 ± 10.72 months)	Median nerve	Cohort A: General Electric Company LEbt12 (5 - 13 MHz linear array transducer)<br>Cohort B: Canon Medical Systems Toshiba Aplio XG (7 - 13 MHz linear array transducer)	To compare the CSA of the median nerve (CSA) between ALS patients and controls and to analyze the role of CSA as a progression and prognostic biomarker.	The CSA is an easy-to-assess biomarker that seems reliable and reproducible. It is also suggested that it could act as a progression and prognostic biomarker in ALS patients.
**Toh et al., 2021**[7]	Malaysia	ALS (40)	ALS (30/10)	NA	ALS (20.5 ± 19.1 months)	Median nerve<br>Ulnar nerve<br>Tibial nerve<br>Fibular nerve	High-resolution NMUS (Mindray 14 MHz M7 with a linear array	To develop a model to predict ALS progression based on clinical and neuromuscular US parameters	The current model is simple and can predict the probability of fast disease progression
**López-Navarro et al., 2021**[8]	Spain	ALS (14)<br>Control (14)	ALS (10/4)<br>Control (10/4)	ALS (68.7 ± 11.57)<br>Control (67.2 ± 12.89)	ALS (19 ± 24.4)	Median nerve<br>Sciatic nerve<br>Common peroneal nerve	Logiq E R8 is equipped with a 12L-RS linear transducer (7?13 MHz)	To determine the behavior of ultrasound biomarkers of fascicle density and muscle strength in patients with ALS.	Muscle thickness measurements derived from dynamic testing and a number of fascicles (NF) and fascicle density (FD) may be useful biomarkers for monitoring patients with ALS and establishing a prognosis.
**Mohamed et al., 2021**[9]	Egypt	ALS (30)<br>Cervical degeneration (30)<br>Control (30)	ALS (21/9)<br>Cervical degeneration (17/13)<br>Control: (19/11)	ALS (43.5 ± 16)<br>Cervical degeneration (52.9 ± 10.2)<br>Control: (42.9 ± 15.7)	ALS (21.6 ± 16.8 months)	Median nerve<br>Ulnar nerve<br>Radial nerve<br>Tibial nerve	Logiq p7 ultrasound machine (GE Healthcare, Waukesha, Wisconsin, USA) with a linear transduced (7–12 MHz)	To evaluate the role of neuro-sonography in the diagnosis and differentiation of amyotrophic lateral sclerosis from other causes of progressive mixed upper and lower motor neuron lesions.	Neurosonography of peripheral nerves is a recent, noninvasive, accessible technique for early diagnosis of ALS.
**Weise et al., 2021**[10]	Germany	ALS (37)<br>Control (40)	ALS (20/17)<br>Control (22/18)	ALS (64.8 ± 11.4)<br>Control (65.7 ± 11.8)	ALS (22.5 ± 23.0 months)	Median nerve<br>Vagus nerve	HRUS using the Esaote MyLab Five system with a 15 MHz transducer (probe LA435)	To investigate the role of the autonomic nervous system in ALS using a multimodal approach	No differences in the vagus nerve or median nerve CSA between healthy controls and patients with ALS were found. No differences were found either after the exclusion of patients with diabetes mellitus
**Holzapfel et al., 2020**[11]	Germany	ALS (24)<br>Control (19)	ALS (16/8)<br>Control (13/6)	ALS (64.04 ± 9.98)<br>Control (63.05 ± 11.07)	ALS (12.46 ± 10.28 Months)	Vagus nerve	Philips IU22 ultrasound machine (17-MHz linear-array transducer)	To find possible sonographic changes of vagus nerve size in bulbar-affected ALS patients.	Bulbar ALS completes the spectrum of disorders associated with vagus nerve atrophy beyond Parkinson's disease and diabetes mellitus.
**Schreiber et al., 2020**[12]	Germany	ALS (177)<br>Control (57)	ALS (107/70)<br>Control (33/24)	ALS (62.3 ± 11.6)<br>Control (59.4 ± 9.7)	ALS (35.6 ± 21.84 months)	Median nerve<br>Ulnar nerve	12-MHz linear-array probe (High-End LOGIQ 7; GE Healthcare, Chicago, Illinois)	To investigate the covariance and disease-specific signature of several sonographic nerve texture features and examined their relationship to the clinical characteristics of ALS patients.	Results point to the potential of sonographic texture markers to assess deep microstructure alterations of the peripheral nerves in ALS.
**Suratos et al., 2020**[13]	Japan	ALS (38)<br>Control (28)	ALS (23/15)<br>Control(10/18)	ALS (64.71 ± 12.76)<br>Control (59.61 ± 24.45)	ALS (23.21 ± 23.11 months)	Phrenic nerve	LOGIQ e Premium device with a 12-MHz linear array	To determine if purely motor phrenic nerve would show sonographic evidence of decreased nerve caliber similar to other peripheral nerves in ALS	This study demonstrates that ALS patients have a smaller PN size compared to controls using ultrasonography
**Deilami et al., 2019**[14]	Iran	ALS (35)<br>Control (35)	ALS (19/16)<br>Control (22/13)	ALS (39.2 ± 14)<br>Control (38 ± 11)	ALS (53.16 ± 13.2 months)	Median nerve	Ultrasonography (SonoSite M-Turbo with an 8-18 Hz linear probe)	To compare the sonographic measurement of median nerve cross-section area (CSA) in patients with Amyotrophic Lateral Sclerosis (ALS) and healthy individuals	CSA was not different between ALS patients and the normal population, but compound muscle action potential (CMAP) decreased in ALS patients. ALS functional rating scale correlated with both CSA and CMAP of the median nerve.
**Díaz et al., 2019**[15]	Spain	ALS (59)<br>Control (20)	ALS (35/24)<br>Control (10/10)	ALS (63.5 ± 10.89)<br>Control (60.2 ± 9.97)	ALS (3.5 ± 6.25 months)	Median nerve	Medical Systems Aplio XG (2008) equipped with a 7-13 MHz phased array transducer	To assess the differences in morphological and texture parameters of median nerve (MN) and abductor pollicis brevis (APB) between amyotrophic lateral sclerosis (ALS) patients and controls.	APB muscle ultrasound biomarkers (especially MTh and EI) showed better discrimination capacity and correlation with clinical variables than MN biomarkers.
**Schreiber et al., 2019**[16]	Germany	ALS training cohort (61)<br>ALS study cohort (112)<br>Control (50)	ALS training cohort (40/21)<br>ALS study cohort (65/47)<br>Control (30/20)	ALS training cohort (63 ± 11)<br>ALS study cohort (61 ± 12)	ALS training cohort (24.49 ± 19.4 months)	Median nerve<br>Ulnar nerve	12-MHZ linear array probe (GE High-End LOGIQ7 system; GE Healthcare, Piscataway, New Jersey)	To identify patients with amyotrophic lateral sclerosis (ALS) who displayed suspected peripheral nervous system (PNS) inflammation to compare them to those with suspected PNS degeneration.	Suspected PNS degeneration was related to classic ALS, shorter disease duration, and a smaller hypoechoic nerve area.

				Control (60 ± 12)	ALS study cohort (23.64 ± 25.0 months)				
**Schreiber et al., 2018[17]**	Germany	ALS (41)<br>Control (18)	ALS (26/15)<br>Control (12/6)	ALS (64 ± 10)<br>Control (59 ± 8)	ALS (34 ± 38 months)	Median nerve<br>Ulnar nerve<br>Radial nerve	12MHz linear array probe (GE High-End LOGIQ7 System).	To assess whether differential peripheral nerve involvement parallels dissociated forearm muscle weakness in amyotrophic lateral sclerosis (ALS).	This systematic study confirmed long-observed physical examination findings in ALS weakness in finger extension out of proportion to finger flexion. This phenomenon was unrelated to any particular sonographic pattern of upper limb peripheral nerve alteration.
**Noto et al., 2018[18]**	Australia	ALS (53)<br>PNHS (23)<br>MMN (9)<br>Control (30)	ALS (41/12)<br>PNHS (15/8)<br>MMN (4/5)<br>Control (20/10)	ALS (59.9 ± 13.9)<br>PNHS (55.4 ± 13.4)<br>MMN (58.0 ± 12.6)<br>Control (59.7 ± 17.6)	ALS (23.0 ± 21.1 months)<br>PNHS (113.5 ± 180.4 months)<br>MMN (140.0 ± 95.5 months)	Median nerve<br>Ulnar nerve<br>Tibial nerve	MyLab Alpha ultrasound machine (Esaote, Genova, Italy) was used with a 6–18 MHZ broadband linear array transducer (SL2325, Esaote)	The aim is to determine the peripheral nerve morphology in patients with ALS and investigate whether variables such as a distal–proximal ratio of CSA may be useful in differentiating ALS from various mimic disorders.	Ultrasound patterns of nerve involvement in ALS and disease mimics have been established. Compared with controls, a decreased median nerve CSA of the upper arm and an increased median nerve CSA wrist–upper arm ratio were the most prevalent findings in ALS.
**Schreiber et al., 2017[19]**	Germany	ALS (55)	ALS (33/22)	ALS (59 ± 13)	ALS (24 ± 21 months)	Median nerve<br>Ulnar nerve	12-MHZ linear array probe (GE High-End LOGIQV R7 System)	To investigate whether sonographic peripheral cross-sectional nerve area (CSA) and progranulin (PGRN) are related to each other and whether they interact to predict clinical and paraclinical measures in amyotrophic lateral sclerosis (ALS).	In an ALS sample, we have demonstrated a relationship between structural peripheral nerve alterations and CNS-derived CSF PGRN.
**Jongbloed et al., 2016[20]**	Netherlands	ALS (10)<br>MMN (10)	ALS (5/5)<br>MMN (5/5)	ALS (54 [11])<br>MMN (55 [6])	ALS (17.74 ± 9.05 months)<br>MMN (58.44 ± 36.53 months)	Median nerve<br>Ulnar nerve	High-resolution ultrasound Philips iU22 (5-MHz to 17-MHz linear array transducer)	To compare the performance of MRI and HRUS of peripheral nerves in the forearm by comparing median and ulnar nerve cross-sectional areas (CSA) in patients with MMN and ALS.	This study shows that peripheral nerve imaging techniques hold promise for improving the diagnostic work-up of patients suspected of having MMN.
**Nodera et al., 2016[21]**	Japan	ALS (12)<br>MMN (9)<br>Control (17)	ALS (15/7)<br>MMN (9/0)<br>Control (11/6)	ALS (60.78 ± 7.85)<br>MMN (44.49 ± 15.06)<br>Control (58.44 ± 15.59)	NA	C5, and C6 nerve roots	High-resolution echo-sonography (Nemio, Toshiba, Japan) using an 11-MHz linear probe.	This study assesses whether sonography of the nerve roots can identify nerve hypertrophy as suggestive evidence of demyelination in MMN patients without conduction block and further helps differentiate MMN from ALS.	Sonography can detect the focal thickness of a cervical nerve root in MMN to suggest demyelination, which can differentiate MMN from ALS, which has been demonstrated to have pathologically thinner nerve roots due to atrophy, and from normal controls.
**Schreiber et al., 2016[22]**	Germany	ALS (37)	ALS (25/12)	ALS (59.6 ± 10.5)	ALS (21.6 ± 22.4 months)	Median nerve<br>Ulnar nerve	12 MHZ linear-array probe (LOGIQ 7 System; GE Healthcare).	To assess whether peripheral nerve sonography could be used as a biomarker to monitor disease progression in amyotrophic lateral sclerosis (ALS)	High-resolution sonography appears to be a valid tool to demonstrate ongoing ulnar nerve atrophy in ALS. The feasibility of the technique in outpatient settings and its tolerability make it particularly suitable for clinical trials.
**Mori et al., 2016[23]**	Japan	ALS (51)<br>Control (37)	ALS (31/20)<br>Control (13/24)	ALS (67.9 ± 10.3)<br>Control (55.3 ± 8.3)	ALS (48.8 ± 188 months)	C5, C6, and C7 nerve roots	LOGIQ7 (GE) with an 11-MHz linear-array transducer	To assess possible motor axon loss in patients with ALS by sonography and to correlate the imaging features with clinical subtypes	There was no correlation between the disease duration and the diameters of the nerve roots. Sonography of the cervical nerve roots showed axonal atrophy in ALS and potentially reflects subtle arm dysfunctions.
**Grimm et al., 2015[24]**	Germany	ALS (17)<br>MMN (8)<br>Control (28)	ALS (9/6)<br>MMN (8/0)<br>Control (17/13)	ALS (65.6 ± 13.3)<br>MMN (55.6 ± 11.2)<br>Control (52.8 ± 17.2)	ALS (18.8 ± 18.3 months)<br>MMN (41.2 ± 21.6 months)	Median nerve<br>Ulnar nerve<br>Tibial nerve<br>Sural nerve<br>Vagus<br>C6 root	14 MHz probe real-time linear array scanner (ZONARE ultrasound systems)	To compare results of CSA measurements in nerves of upper and lower extremities, vagus nerve, and the sixth cervical nerve root in both ALS and MMN and, secondly, to determine the role of PNUS in differentiating between these two conditions.	Systematic ultrasound measurements in different nerves and nerve roots are valuable for detecting focal nerve enlargement in MMN, which is generally not found in ALS. Thus, they could serve as a diagnostic marker to differentiate between both entities in addition to electrodiagnostic studies.
**Loewenbrück et al., 2015[25]**	Germany	Cohort 1<br>ALS (16)<br>MMN (8)<br>Cohort 2<br>ALS (8)<br>MMN (5)<br>Other (17)	Cohort 1<br>ALS (12/4)<br>MMN (6/2)<br>Cohort 2<br>ALS (2/6)<br>MMN (3/12)<br>Other (0/17)	Cohort 1<br>ALS (58.7 ± 8.2)<br>MMN (69.6 ± 7.5)<br>Cohort 2<br>ALS (65.1 ± 8.8)<br>MMN (53.6 ± 11.7)<br>Other (60.3 ± 14.7)	Cohort 1<br>ALS (22 ± 14 months)<br>MMN (56 ± 27 months)	Median nerve<br>Ulnar nerve<br>Radial nerve<br>C5, C6, C7 roots<br>Vagus nerve<br>Sciatic nerve<br>Tibial nerve	Aplio MX, linear transducer, 8 to 18 MHz (Toshiba, Neuss, Germany) and MyLab Five, linear transducer, 10 to 18 MHz (Esaote Biomedica, Cologne, Germany)	To investigate nerve ultrasound in comparison to nerve conduction studies for differential diagnosis of ALS/LMND and MMN.	Nerve US is of high diagnostic accuracy for differential diagnosis of ALS/LMND and MMN. It might be superior to NCS in diagnosing MMN in hospital-admitted patients with this differential diagnosis.
**Mori et al., 2014[26]**	Japan	ALS (21)<br>CTS (14)<br>Control (30)	ALS (17/4)<br>CTS (8/6)<br>Control (20/10)	ALS (68.3 ± 9.1)<br>CTS (63.9 ± 16.8)<br>Control (61.8 ± 18.1)	NA	Median nerve<br>Ulnar nerve	LOGIQ7 (GE) with an 11-MHz linear-array transducer	To depict the electrophysiologic and sonographic characteristics of the median nerve at the wrist that could elucidate the pathophysiology of ALS.	Selective conduction slowing of the median nerve at the wrist in ALS is unlikely due to secondary compressive neuropathy, as seen in carpal tunnel syndrome.
**Nodera et al., 2014[27]**	Japan	ALS (35)<br>Control (37)	ALS (20/15)<br>Control (13/24)	ALS (58.0 ± 11.5)<br>Control (55.3 ± 8.3)	ALS (34.8 ± 24 months)	Median nerve<br>Ulnar nerve<br>C5, C6, and C7 nerve roots	LOGIQ7 (GE) with an 11-MHz linear-array transducer	To evaluate the cervical nerve roots and peripheral nerves in patients with ALS to find whether pathologically proven thinning of peripheral nerves and nerve roots can be identified in vivo by sonography.	Cervical nerve roots might be more appropriate for detecting motor axon loss than peripheral nerves.
**Schreiber et al., 2014[28]**	Germany	PLS (8)<br>UMND (14)<br>LMND (20)<br>Classic (21)<br>Bulbar (15)<br>Control (18)	PLS(5/3)<br>UMND(11/3)<br>LMND(14/6)<br>Classic(12/9)<br>Bulbar(6/9)<br>Controls(14/4)	PLS (63.5 ± 16.8)<br>UMND (54.1 ± 8.3)<br>LMND (59.2 ± 14.5)<br>Classic (58.5 ±12.1)<br>Bulbar (66.1 ± 11.4)<br>Control (63.8 ± 9.5)	PLS (104.1 ± 77.7 months)<br>UMND (32.8 ± 22.4 months)<br>LMND (37.0 ± 26.0 months)<br>Classic (20.9 ± 12.8 months)<br>Bulbar (15.7 ± 5.7 months)	Median nerve<br>Ulnar nerve	(LOGIQ7; GE Healthcare) with a12-MHZ linear array probe	To determine the cross-sectional area (CSA) of peripheral nerves in patients with distinct amyotrophic lateral sclerosis (ALS) subtypes.	Ulnar nerve ultrasound in ALS subgroups revealed significant differences in distal CSA values, which suggests it has value as a marker of LMN involvement. Its potential was particularly evident in the UMND and PLS groups, which can be hard to separate clinically, yet their accurate separation has major prognostic implications.
**Cartwright et al., 2011[29]**	USA	ALS (20)<br>Control (20)	ALS (10/10)<br>Control (10/10)	ALS (57.5 ± 8.3)<br>Control (58.4 ± 9.1)	ALS (25.1 months)	Median nerve<br>Sural nerve	High-resolution US (18-MHz linear-array transducer)	To determine whether neuromuscular ultrasound revealed peripheral nerve or muscle abnormalities obvious enough to assist in diagnosing ALS.	Neuromuscular ultrasound demonstrates nerve and muscle atrophy in ALS and should be further explored as a disease biomarker.

Quality assessment

Case-control studies by The Newcastle-Ottawa Scale (NOS) quality assessment[30]

Table S3. Case-control studies by The Newcastle-Ottawa Scale (NOS) quality assessment

Study	Selection of case and controls				Comparability of cases and controls	Exposure			overall	Quality
	Is the case definition adequate	Representativeness of the cases	Selection of controls	Definition of controls	Comparability of cases and controls on the basis of the design or analysis	Assortment of exposure	The same method of ascertainment for case and controls	Non-Response rate		
**Laucius et al., 2023[1]**	*	*	*	*	**	*	*	*	9	Fair
**Fan et al., 2023[2]**	*	*	*	*	**	*	*	/	8	Good
**Martínez-Payá et al.,2022[6]**	*	*	/	*	**	*	*	/	7	Good
**Weise et al., 2021[10]**	*	*	/	*	- -	*	*	/	5	Fair
**Mohamed et al., 2021[9]**	*	*	*	*	**	*	*	-	8	Fair
**Holzapfel et al., 2020[11]**	*	*	*	*	**	-	*	-	7	Fair
**Noto et al., 2018[18]**	*	*	*	*	- -	/	*	-	5	Fair

Cohort/cross-sectional studies by NIH[31]

Table S4. Quality assessment of Cohort/cross-sectional studies by NIH

Study name	N1	N2	N3	N4	N5	N6	N7	N8	N9	N10	N11	N12	N13	N14	Total	Quality
**Hildebrand et al., 2023[3]**	*	*	-	*	/	*	*	*	*	*	*	-	-	-	9	Good
**Walter et al., 2023[4]**	*	*	/	*	-	-	-	*	*	-	*	*	*	*	9	Good
**Watanabe et al., 2022[5]**	*	*	/	*	-	-	-	*	*	-	*	-	*	-	7	Good
**López-Navarro et al., 2021[8]**	*	*	*	*	-	/	/	/	/	/	*	*	*	*	8	Good
**Toh et al., 2021[7]**	*	*	/	*	-	*	*	/	/	-	*	*	*	*	9	Good
**Schreiber et al., 2020[16]**	*	*	/	*	*	*	*	/	*	/	*	-	/	*	9	Good
**Suratos et al., 2020[13]**	*	*	/	*	-	/	/	/	/	-	*	-	/	*	5	Fair
**Deilami et al., 2019[14]**	*	-	*	*	-	*	*	*	*	-	*	*	*	*	11	Good
**Schreiber et al., 2019[17]**	*	*	/	*	-	/	/	/	*	-	*	-	/	*	6	Fair
**Ríos-Díaz et al., 2018[15]**	*	*	/	*	/	*	*	*	*	*	*	*	/	*	11	Good
**Schreiber et al., 2018[17]**	*	*	/	*	-	*	*	/	*	/	*	-	/	*	8	Good
**Schreiber et al., 2017[19]**	*	*	/	-	-	*	*	-	*	/	*	-	*	*	8	Good
**Schreiber et al., 2016[22]**	*	*	/	-	-	*	*	*	*	/	*	*	*	*	10	Good
**Nodera et al., 2016[21]**	*	*	/	*	-	*	/	*	*	/	*	*	*	-	9	Good
**Mori et al.,2016[23]**	*	*	/	-	-	*	/	/	*	-	*	-	*	*	7	Fair
**Jongbloed et al., 2016[20]**	*	*	-	*	-	*	*	*	*	*	*	-	-	-	9	Good
**Grimm et al., 2015[24]**	*	*	*	*	-	*	*	/	*	*	*	*	*	-	11	Good
**Loewenbruck et al., 2015[25]**	*	*	*	*	-	/	/	/	/	/	*	*	*	*	8	Good
**Schreiber et al., 2014[28]**	*	*	/	-	-	*	*	*	*	/	*	-	*	*	9	Good
**Mori et al.,2014[26]**	*	*	/	-	-	*	/	*	*	-	*	*	*	*	9	Good
**Nodera et al., 2014[27]**	*	*	/	*	/	*	*	*	*	*	*	/	/	/	9	Good
**Cartwright et al., 2011[29]**	*	-	*	*	-	*	*	/	*	*	*	-	*	-	9	Good

*Yes -No

/ Cannot determine, not applicable, not reported

Quality > Fair (less than 8) > Good (=8 or more)

N1: Was the research question or objective in this paper clearly stated?

N2: Was the study population clearly specified and defined?

N3: Was the participation rate of eligible persons at least 50%?

N4: Were all the subjects selected or recruited from the same or similar populations (including the same time period)? Were inclusion and exclusion criteria for being in the study prespecified and applied uniformly to all participants?

N5: Was a sample size justification, power description, or variance and effect estimates provided?

N6: For the analyses in this paper, were the exposure(s) of interest measured prior to the outcome(s) being measured?

N7: Was the timeframe sufficient so that one could reasonably expect to see an association between exposure and outcome if it existed?

N8: For exposures that can vary in amount or level, did the study examine different levels of the exposure as related to the outcome (e.g., categories of exposure, or exposure measured as continuous variable)?

N9: Were the exposure measures (independent variables) clearly defined, valid, reliable, and implemented consistently across all study participants?

N10: Was the exposure(s) assessed more than once over time?

N11: Were the outcome measures (dependent variables) clearly defined, valid, reliable, and implemented consistently across all study participants?

N12: Were the outcome assessors blinded to the exposure status of participants?

N13: Was loss to follow-up after baseline 20% or less?

N14: Were key potential confounding variables measured and adjusted statistically for their impact on the relationship between exposure(s) and outcome(s)?

Figures of Leave out Meta-analysis plots

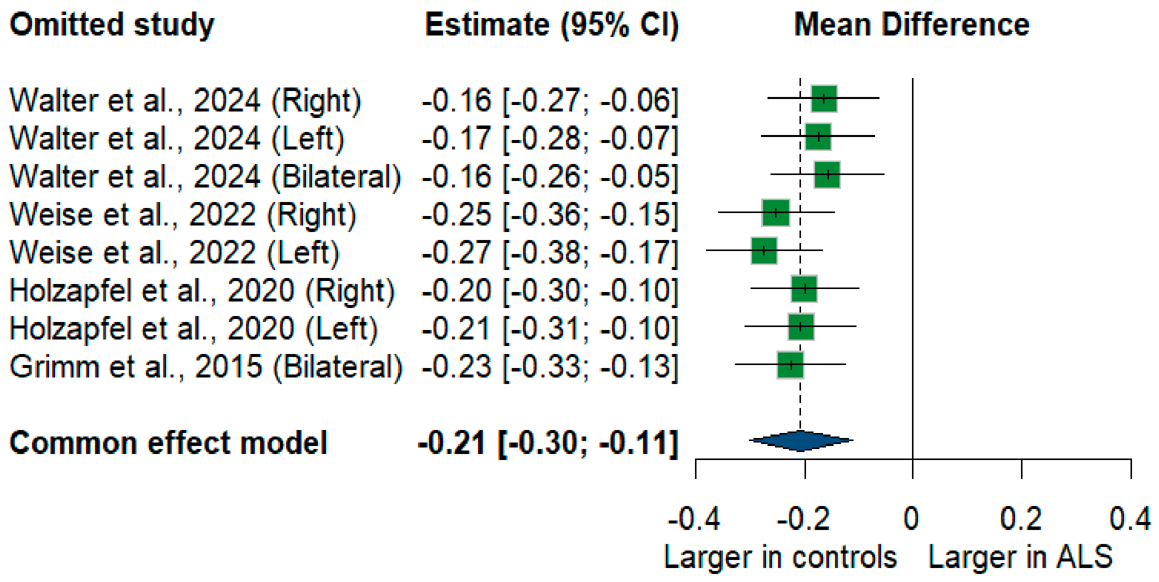

Figure S1. Leave one out meta-analysis for All Vagus nerve measurements. CI=confidence interval; ALS=Amyotrophic lateral sclerosis

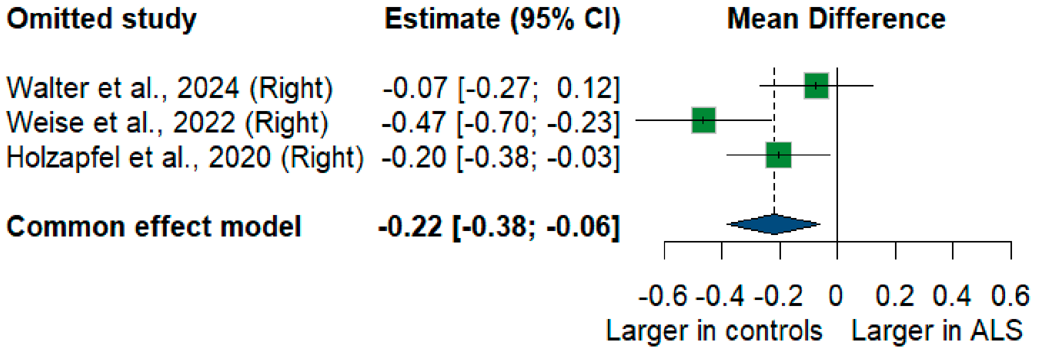

Figure S2. Leave one out meta-analysis for right vagus nerve measurements. CI=confidence interval; ALS=Amyotrophic lateral sclerosis

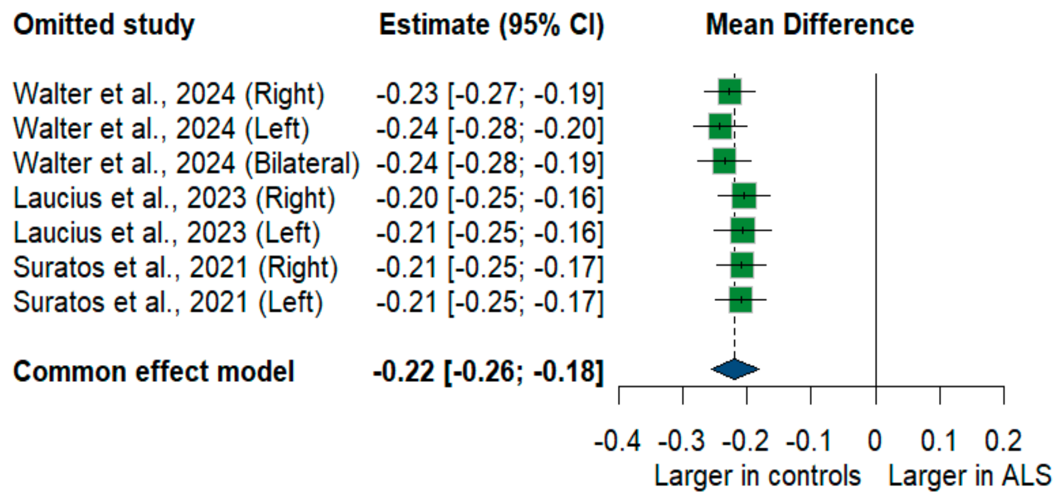

Figure S3. Leave one out meta-analysis for All Phrenic nerve measurements. CI=confidence interval; ALS=Amyotrophic lateral sclerosis

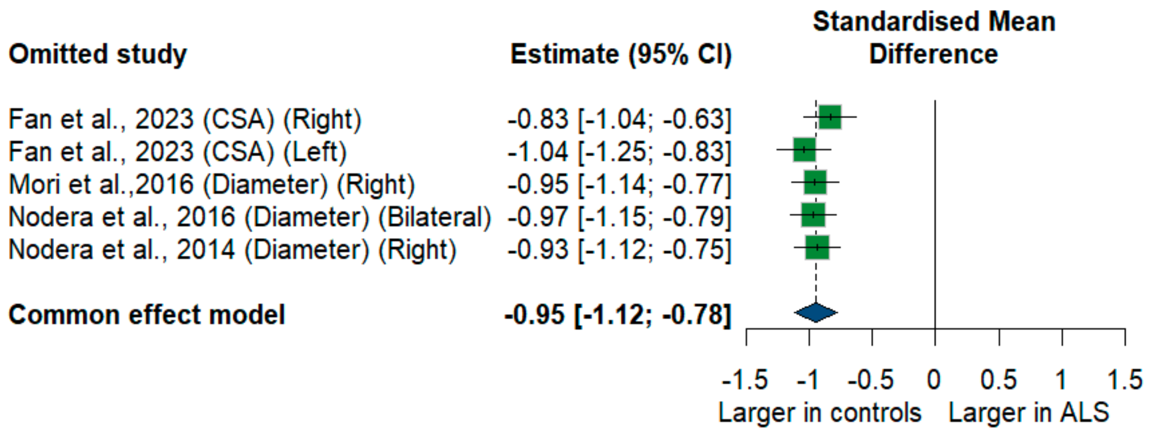

Figure S4. Leave one out meta-analysis for All C5 root measurements. CI=confidence interval; CSA=Cross sectional area; ALS=Amyotrophic lateral sclerosis

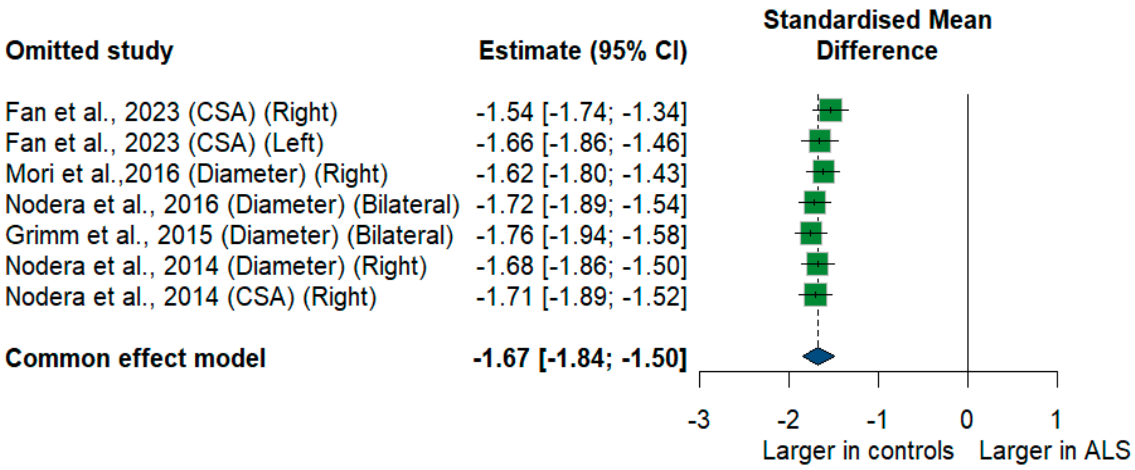

Figure S5. Leave one out meta-analysis for All C6 root measurements. CI=confidence interval; CSA=Cross sectional area; ALS=Amyotrophic lateral sclerosis

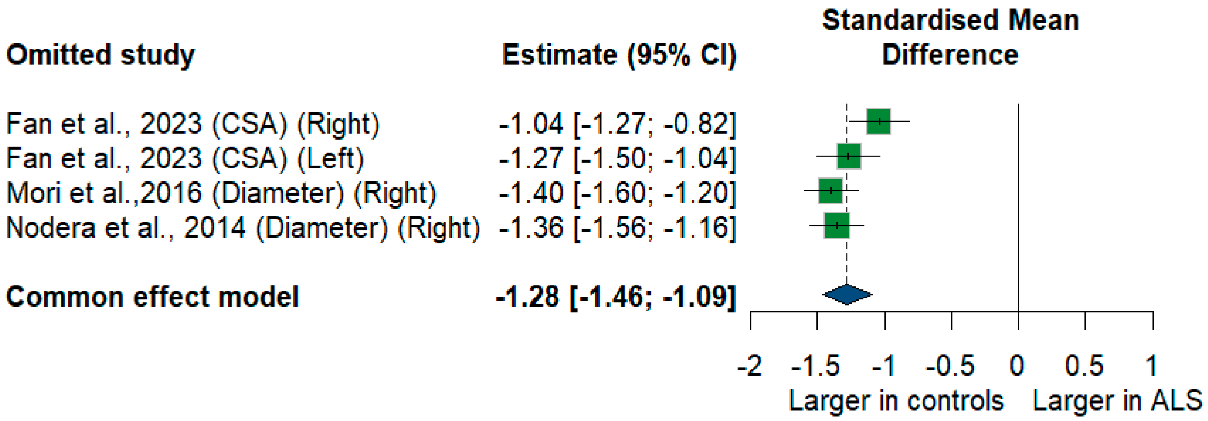

Figure S6. Leave one out meta-analysis for All C7 root measurements. CI=confidence interval; CSA=Cross sectional area; ALS=Amyotrophic lateral sclerosis

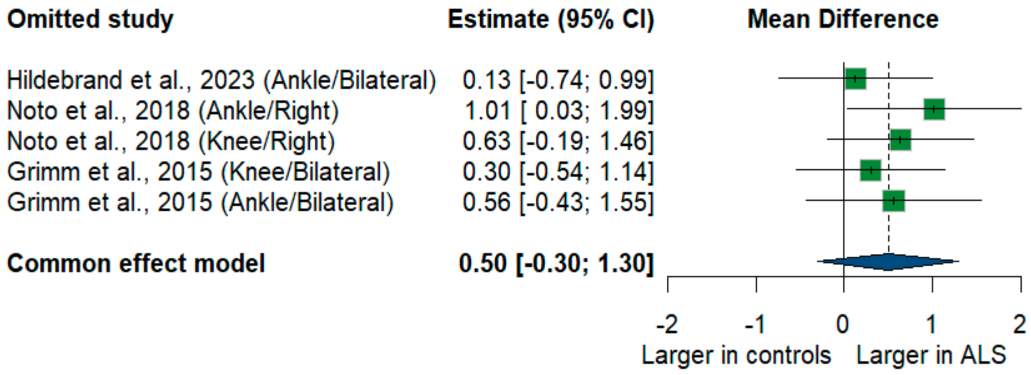

Figure S7 .Leave one out meta-analysis for All Tibial nerve measurements. CI=confidence interval; ALS=Amyotrophic lateral sclerosis

Omitted study	Estimate (95% CI)
Hildebrand et al., 2023 (Ankle/Bilateral)	15.70 [11.46; 19.93]
Toh et al., 2021 (Knee/Right)	13.91 [10.97; 16.85]
Toh et al., 2021 (Ankle/Right)	15.22 [10.94; 19.51]
Mohamed et al.,2021 (Ankle/Right)	15.88 [11.73; 20.03]
Mohamed et al.,2021 (Knee/Right)	15.50 [11.22; 19.79]
Mohamed et al.,2021 (Ankle/Left)	15.85 [11.69; 20.02]
Mohamed et al.,2021 (Knee/Left)	15.48 [11.19; 19.77]
Noto et al., 2018 (Ankle/Right)	15.54 [11.25; 19.82]
Noto et al., 2018 (Knee/Right)	14.75 [10.68; 18.83]
Loewenbruck et al., 2016 (Ankle/Bilateral)	16.02 [11.96; 20.08]
Grimm et al., 2015 (Knee/Bilateral)	14.66 [10.66; 18.66]
Grimm et al., 2015 (Ankle/Bilateral)	15.90 [11.77; 20.04]
**Random effects model**	**15.37 [11.45; 19.28]**

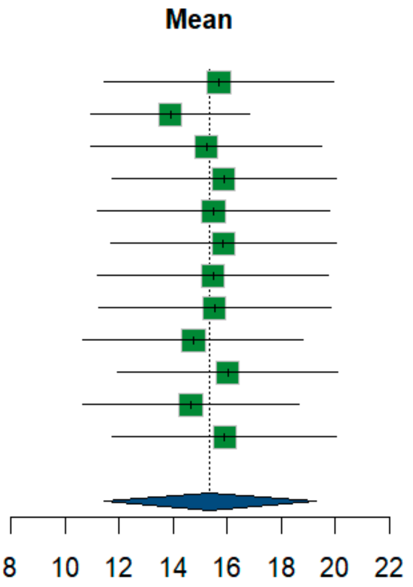

Figure S8. Leave one out meta-analysis for single arm Tibial nerve measurements. CI=confidence interval; ALS=Amyotrophic lateral sclerosis

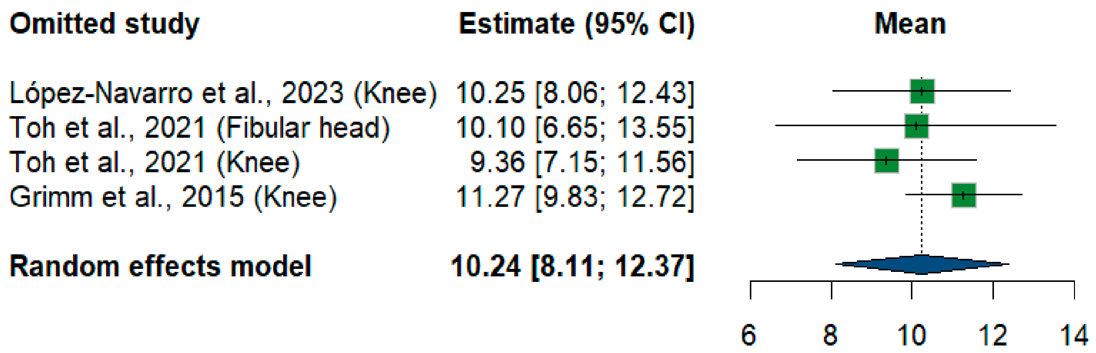

Figure S9. Leave one out meta-analysis for single arm Fibular nerve measurements. CI=confidence interval; ALS=Amyotrophic lateral sclerosis

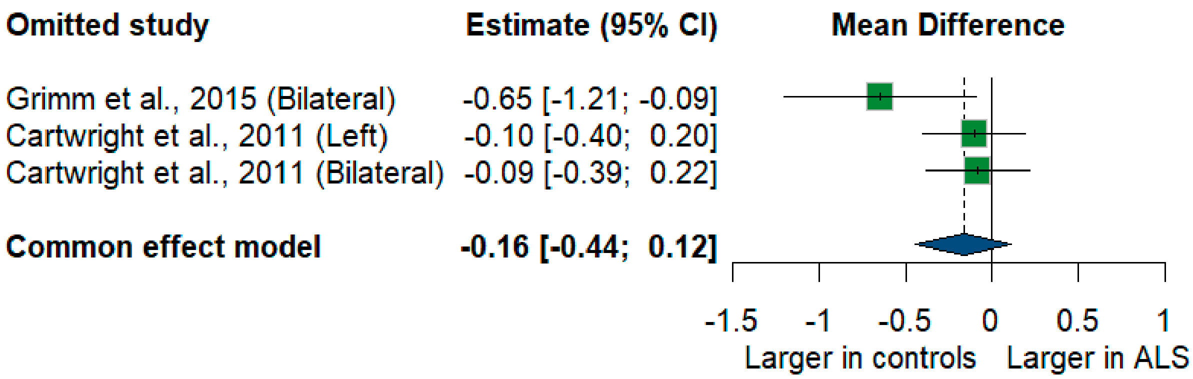

Figure S10. Leave one out meta-analysis for All Sural nerve measurements. CI=confidence interval; ALS=Amyotrophic lateral sclerosis

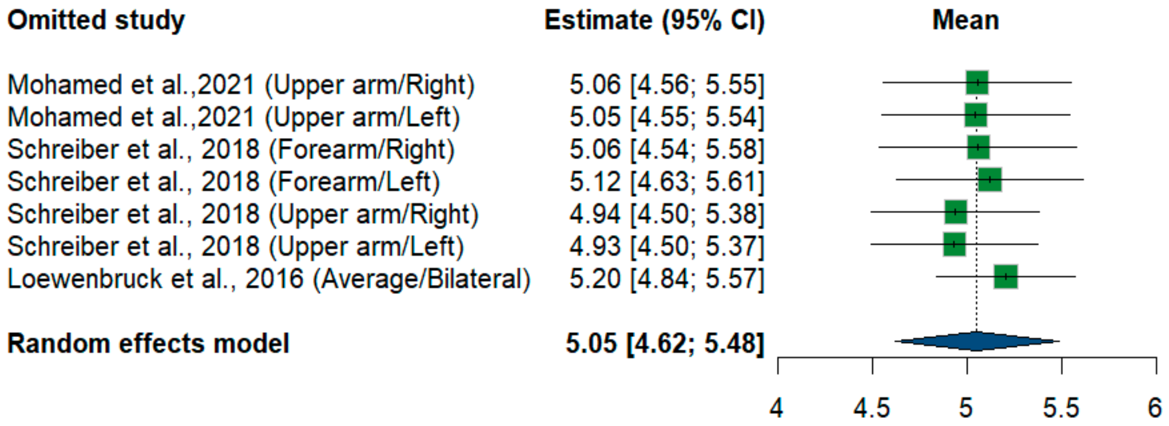

Figure S11. Leave one out of the meta-analysis for single-arm Radial nerve measurements. CI=confidence interval; ALS=Amyotrophic lateral sclerosis

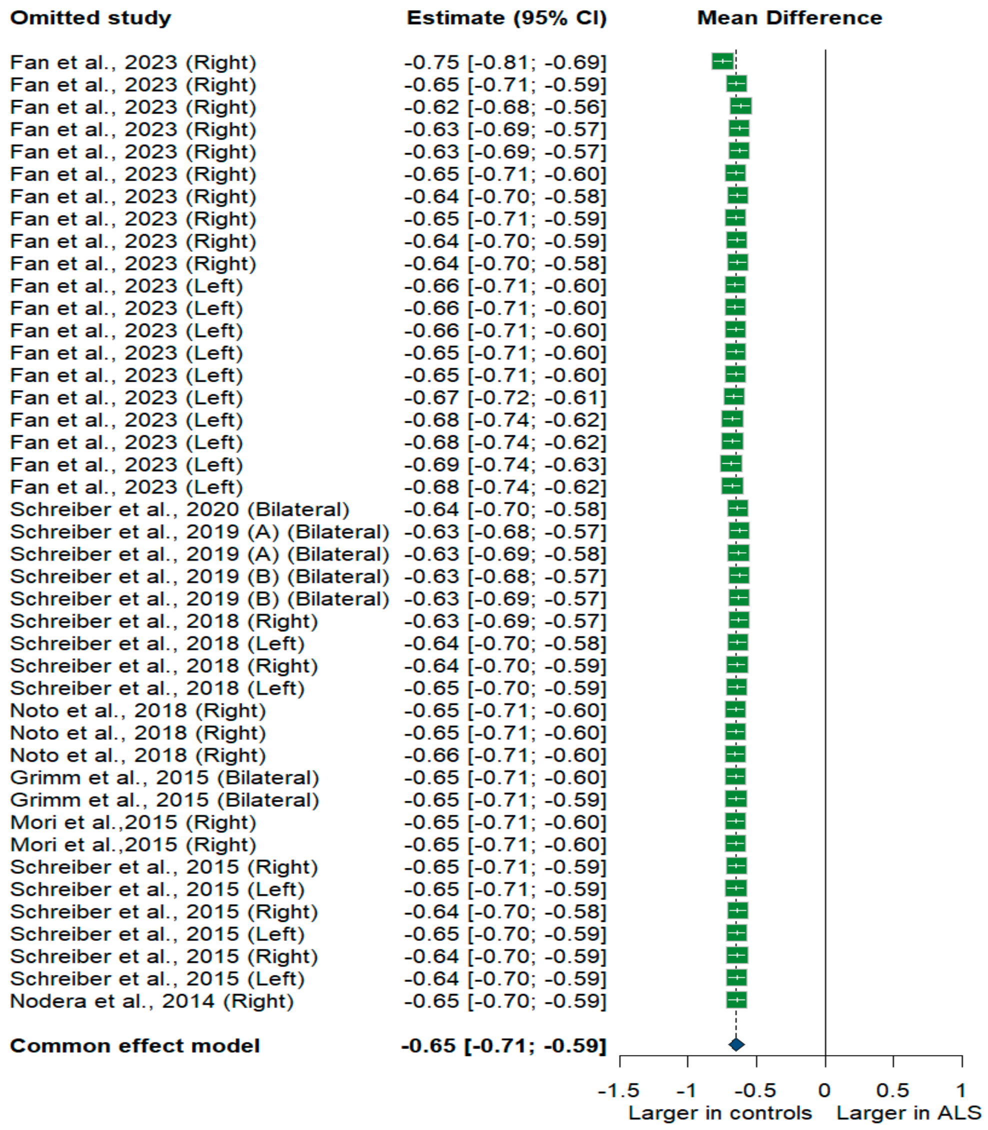

Figure S12. Leave one out meta-analysis for All Ulnar nerve measurements. CI=confidence interval; ALS=Amyotrophic lateral sclerosis

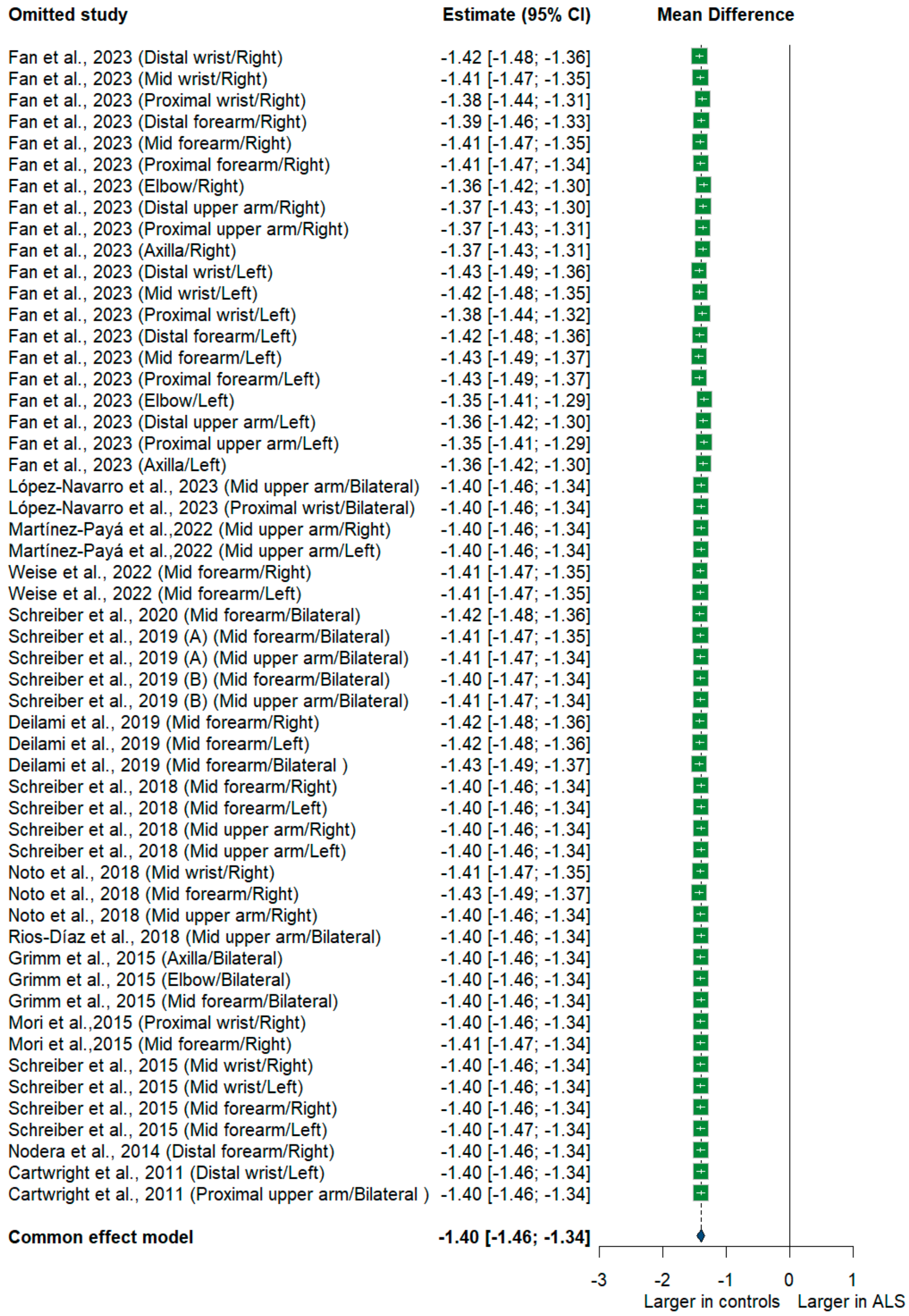

Figure S13. Leave one out meta-analysis for All Median nerve measurements. CI=confidence interval; ALS=Amyotrophic lateral sclerosis
